# Chronic rapid eye movement sleep deprivation aggravates the pathogenesis of Alzheimer’s disease by decreasing brain *O*-GlcNAc cycling in mice

**DOI:** 10.1186/s12974-024-03179-4

**Published:** 2024-07-23

**Authors:** Dong Yeol Kim, Sang-Min Kim, Inn-Oc Han

**Affiliations:** Department of Biomedical Science, Program in Biomedical Science and Engineering, Department of Physiology and Biophysics, College of Medicine, Inha University, Incheon, Korea

**Keywords:** REM sleep, Hexosamine, Cognition, Alzheimer’s disease, *O*-GlcNAc transferase

## Abstract

This study investigated the role of *O*-GlcNAc cycling in Alzheimer’s disease-related changes in brain pathophysiology induced by chronic REM sleep deprivation (CSD) in mice. CSD increased amyloid beta (Aβ) and p-Tau accumulation and impaired learning and memory (L/M) function. CSD decreased dendritic length and spine density. CSD also increased the intensity of postsynaptic density protein-95 (PSD-95) staining. All of these Alzheimer’s disease (AD) pathogenic changes were effectively reversed through glucosamine (GlcN) treatment by enhancing O-GlcNAcylation. Interestingly, the lelvel of *O*-GlcNAcylated-Tau (*O*-Tau) exhibited an opposite trend compared to p-Tau, as it was elevated by CSD and suppressed by GlcN treatment. CSD increased neuroinflammation, as indicated by elevated levels of glial fibrillary acidic protein and IBA-1-positive glial cells in the brain, which were suppressed by GlcN treatment. CSD promoted the phosphorylation of GSK3β and led to an upregulation in the expression of endoplasmic reticulum (ER) stress regulatory proteins and genes. These alterations were effectively suppressed by GlcN treatment. Minocycline not only suppressed neuroinflammation induced by CSD, but it also rescued the decrease in *O*-GlcNAc levels caused by CSD. Minocycline also reduced AD neuropathy without affecting CSD-induced ER stress. Notably, overexpressing *O*-GlcNAc transferase in the dentate gyrus region of the mouse brain rescued CSD-induced cognitive dysfunction, neuropathy, neuroinflammation, and ER stress responses. Collectively, our findings reveal that dysregulation of *O*-GlcNAc cycling underlies CSD-induced AD pathology and demonstrate that restoration of *O*GlcNAcylation protects against CSD-induced neurodegeneration.

**Supplementary Information:**

The online version contains supplementary material available at 10.1186/s12974-024-03179-4.

## Introduction

Sleep can be categorized into two distinct stages—REM (rapid eye movement) sleep and non-REM sleep—with the former characterized, in part, by its shorter duration. Sleep deprivation (SD), particularly deprivation of REM sleep (REMSD), can have a significant impact on mood, learning and memory (L/M) function, and may even contribute to the development of neuropsychiatric disorders, including Alzheimer’s disease (AD) [1–3], Parkinson’s disease [4], and depression [5]. SD can be further classified based on its duration. Acute SD refers to continuous SD lasting from several hours to a week, whereas chronic SD involves SD for several hours that persists for weeks or months. Both acute and chronic SD are associated with cognitive impairments and neurodegenerative disorders, but chronic SD in particular has been specifically implicated in the pathogenesis of AD. Indeed, chronic insomnia is a known risk factor for accelerated cognitive decline with aging [6] and older adults experiencing chronic sleep disorders are more likely to exhibit rapid cognitive decline and have an increased risk of developing AD [7].

Prolonged SD has been reported to decrease glucose uptake by 10–20% in the brains of humans and rodents [8, 9]. Multiple lines of evidence suggest that disrupted sleep is likely to impair neurometabolism, particularly in the hippocampus—a brain region known to be involved in L/M processes [10–12]. Furthermore, alterations in brain glucose metabolism are frequently observed before the manifestation of clinical symptoms of dementia, and several studies have demonstrated a reduction in glucose uptake in the brains of patients with AD [13, 14]. Therefore, it can be hypothesized that prolonged disruption in glucose metabolism during chronic SD may be a significant contributing factor to the development of cognitive dysfunction and AD. However, the mechanisms by which abnormalities in brain glucose metabolism contribute to cognitive impairment or AD are still largely unknown. In our previous research, we proposed a significant association between dysfunction in the hexosamine biosynthetic pathway (HBP), which accounts for 2–5% of glucose metabolism [15], and cognitive impairment induced by acute SD in both mouse and zebrafish [16, 17]. The HBP produces UDP-N-acetylglucosamine (UDP-GlcNAc), which serves as a substrate for *O*-linked-GlcNAc modification (*O*-GlcNAcylation). *O*-GlcNAc transferase (OGT) catalyzes the transfer of a single moiety of GlcNAc from UDP-GlcNAc to the hydroxyl side chains of serine and threonine residues on target proteins [18], whereas *O*-GlcNAcase (OGA) reverses this process by catalyzing the removal of *O*-GlcNAc from proteins [18]. The activity of OGT is responsive to the levels of its substrate, UDP-GlcNAc, which in turn are influenced by changes in intracellular glucose metabolism. *O*-GlcNAcylation has recently drawn the attention of researchers in the neuroscience community, reflecting the fact that multiple neuronal proteins undergo *O*-GlcNAcylation at various sites—a process that has the potential to contribute to various neurological disorders [19]. Notably, the presence of *O*-GlcNAc modifications on Tau and amyloid precursor protein (APP), both of which play crucial roles in the development of AD, has sparked interest in exploring the involvement of *O*-GlcNAc in the pathogenesis of AD. Although researchers are only beginning to investigate the role and mechanisms of HBP/*O*-GlcNAc cycling in sleep-related brain dysfunction and AD, emerging evidence suggests that dysregulation of *O*-GlcNAc cycling is critically involved in the development and progression of various neurodegenerative brain disorders induced by sleep disturbance.

In this study, we established a chronic murine SD model in which mice were subjected to REMSD followed by a recovery period under normal light-dark conditions. This cycle of SD and recovery was repeated over a period of 28 days. CSD model mice exhibited cognitive impairments together with pathological features associated with AD. Using this model, we analyzed the reversible and cyclical alterations in OGT, OGA, and *O*-GlcNAcylation in the hippocampus in response to SD cycles. Our results provide compelling evidence that *O*-GlcNAc cycling plays a pivotal role in the development of cognitive impairment, neurodegeneration, neuroinflammation, and AD pathogenesis. These novel findings offer strong evidence supporting the previously hypothesized relationships between sleep, cognitive impairment, and *O*-GlcNAc cycling.

## Materials and methods

### Cell culture

Neuro2a mouse neuroblastoma cell line (Korean Cell Bank, Seoul, Korea), obtained from the Korean Cell Bank in Seoul, Korea, was used at passage numbers 2 to 6. Neuro2a cells were maintained at 37℃ at 5% CO_2_ in DMEM supplemented with 10% fetal bovine serum (Hyclone, UT, USA), 1% (v/v) streptomycin, and penicillin.

### Chronic REM sleep deprivation (CSD) mouse model

Male C57BL/6 N mice, aged 7 to 8 weeks and weighing between 24 and 26 g, were obtained from SAMTACO (Osan, South Korea). The mice were housed individually in cages (15 cm x 30 cm), and they were maintained under a 12-hrs light-dark cycle at a temperature of 22 ± 2℃. All animal experiments conducted in this study were approved by the Institutional Animal Care and Use Committee of Inha University in Incheon, Korea (INHA 190920-665).

REMSD was created using a modified multiple platform method, as described in previous studies [17]. Chronic REMSD (CSD) was induced by transferring mice to a separate holding area filled to a height of 1 cm with water (maintained at a temperature of 23 ± 2 °C). The water tank used in the study had dimensions of 15 × 30 cm and was equipped with 12 cylindrical platforms, each with a diameter of 3.5 cm and a height of 3 cm, on which mice were placed. In this setup, mice were motivated to avoid the water, leading to disrupted sleep and an inability to maintain a stable resting position. Before the experiments, mice were positioned on the platforms and learned to stay on the platform for 30–60 min. Mice in the sleep-deprived group were placed on the platforms for three consecutive days, followed by a 4-day period in their normal cages during which sleep was restored. This cycle of SD and restoration was repeated a total of 4 times over a period of 28 days. GlcN (200 mg/kg) and minocycline (10 mg/kg) were administered intraperitoneally three times a week during sleep deprivation cycle. One day after the final sleep-deprivation cycle, passive avoidance tests and novel object recognition tests (NOR) were administered. All mice were provided ad libitum access to water and food throughout the study.

### Passive avoidance test

The passive avoidance or step-through test was conducted using a system (Jeungdo Bio&Plant Co, Ltd., Seoul, Korea) consisting of light and dark compartments separated by a door [17]. During the habituation stage, the mice were placed in a box and allowed to explore the two compartments for a period of 10 min. For the test phase, each mouse was initially placed in the light chamber of the system, and then the door separating the light and dark chambers was opened, allowing the mouse step through to the dark chamber. After the mice entered the dark chamber, the door was promptly closed, and a foot shock of 0.7 mA for 1 s was administered to the mice. Learning sessions consisted of 3 trials, with each trial being separated by 1-hr intervals. A memory retention test was conducted on the day following the second, third, and fourth weeks using the same parameters, but without administering a foot shock. Memory retention was assessed by measuring the step-through latency, with a maximum measurement time of 300 s.

### Novel object recognition test (NOR)

The novel object recognition (NOR) test was performed following the previously described protocol [20]. NOR test involved a familiarization session and a test session in an open-field arena made of acrylic with dimensions of 30 × 30 × 30 cm, containing 2 objects. The mice were given 10 min to freely explore two identical objects, after which they were returned to their home cages. After 24 h, the animals were reintroduced to the apparatus, where they encountered one of the objects from the familiarization session and a novel object. The difference in the amount of time spent exploring the new object compared to the familiar object indicates the animal’s memory of the previous experience and their learning abilities. The discrimination index (DI), which represents the preference for the new object over the familiar object, can be calculated using the formula: DI = (time of novel one - time of familiar one) / (time of novel one + time of familiar one).

### RNA extraction and real-time polymerase chain reaction (RT-PCR)

Total RNA was extracted from cells using TRIzol™ (Invitrogen, CA, USA) following the manufacturer’s protocol. Complementary DNA (cDNA) was synthesized from 1 µg of RNA using GoScript Reverse Transcriptase (Promega, WI, USA) according to the manufacturer’s instructions. PCR was performed using mouse-specific primers for each target (GRP78, F: GGTGCAGCAGGACATCAAGTT, R: CCCACCTCCAATATCAACTTGA; GRP94, F: AATAGAAAGAATGCTTCGCC, R: TCTTCAGGCTCTTCTTCTGG; CHOP, F: CTGCCTTTCACCTTGGAGAC, R: CGTTTCCTGGGGATGAGATA, GAPDH, F: TCATTGACCTCAACTACAGGT, R: CTAAGCAGTTGGTGGTGCAG). The cDNAs were amplified using SYBR Green Real-time PCR Master Mix (TOYOBO, Osaka, Japan). The expression levels were normalized to the expression of GAPDH.

### Mouse brain tissue preparation and immunofluorescence

The isolated brains were processed for immunohistochemistry following previously described methods [17]. In brief, the brains were fixed in 4% paraformaldehyde in 0.1 M PBS (pH 7.4) for 20 min. Subsequently, the brains were transferred to a fresh solution of 4% paraformaldehyde and kept in this solution for 7 days. The brains were then embedded in paraffin and sectioned using a microtome. Serial coronal sections of the mouse brain were made at -1.06, -1.76, and − 2.06 mm relative to bregma using the microtome (HISTOCUT 820, Leica, Wetzlar, Germany) [21]. For immunofluorescent staining, paraffin sections of the brain were deparaffinized. The sections were then washed with 0.1% PBS-T (2.7 mM KCl, 10 mM Na_2_HPO_4_, 137 mM NaCl, 1.8 mM KH_2_PO_4_ and 0.1% Tween 20; pH 7.6) and blocked in 10% normal goat serum (Jackson ImmunoResearch, PA, USA) in 0.1% PBS-T. Subsequently, the sections were incubated in 1% PBS-T containing the following antibodies: anti-RL-2 (MA1-072, 1:100, Invitrogen), anti-OGT (1:100, sc-74,546, Santa Cruz Biotechnology, CA, USA), anti-MAP-2 (sc-74,421, 1:00, Santa Cruz Biotechnology, CA, USA), anti-OGA (14711-1-AP, 1:100, Proteintech, IL, USA), anti-p-Tau (S231, ab194815, 1:100, Abcam, Cambridge, UK), anti-PSD-95 (ab18258, 1:100, Abcam, Cambridge, UK), anti-GlcNAc-Tau (AS55945, 1:100, Anaspec, TX, USA), anti-Amyloid β (1:100, NBP2-13075, Novos Biologicals), anti-IBA-1 (ab178846, 1:100, Abcam, Cambridge, UK), and GFAP (G3893, 1:100, Sigma), and incubated overnight at 4 °C. After washing, the slides were incubated with a mixture of secondary antibodies, including goat anti-mouse or rabbit IgG conjugated to Alexa 488 or Alexa 564 (Invitrogen, CA, USA). Cell counterstaining was performed using DAPI (Sigma) to visualize the nuclei. The slides were then examined under a confocal LSM 510 META microscope (Carl Zeiss, Jena, Germany) and analyzed using ZEN Light Edition software.

### Golgi-cox staining

Golgi-Cox staining was performed following the previously described method [17]. Mice were euthanized, and isolated brains were immersed in Golgi-Cox staining solution (100 ml D.W, 5% potassium dichromate, 50 ml 5% mercuric chloride, and 40 ml 5% potassium chromate) for 8 days at RT in the dark. After washing with D.W., samples were transferred to tissue protectant buffer and maintained at 4 ℃ in the dark for 8 days. Coronal Sect. (100 μm) were then obtained using a vibratome (VT1000S, Leica, Germany) to generate slides. The slides were subjected to a series of developing steps involving gradient ethanol dehydration with 5% sodium thiosulfate. Subsequently, images were captured using an Olympus IX 83 light microscope (Olympus, Tokyo, Japan) and analyzed using Cellsens Dimension software (Olympus) and ImageJ software (NIH) with a 60x objective oil immersion lens. Neurons were randomly selected for examination of dendrite length and spine density. The length of each dendrite and the spine density were measured using Fiji ImageJ software, following previously described methods [22].

### Western blotting

The whole hippocampus was homogenized in RIPA buffer (1% Na-deoxycholate, 1% NP-40, 150 mM NaCl, 1 mM EDTA, 10 mM Tris pH 7.4) supplemented with protease and phosphatase inhibitors, 20 mM streptozotocin, and incubated for 2 h on ice. After incubation, the samples were centrifuged for 15 min at 13,200 rpm. Protein samples (20 µg) were separated by SDS-PAGE and transferred onto Amersham Biosciences™ Protan™ nitrocellulose membranes (GE Healthcare Life Sciences, Germany). The membranes were incubated with 5% BSA or 5% skim milk in TBS-T for 1 h at RT, followed by incubation with primary antibodies against RL-2 (MA1-072, 1:100, Invitrogen), anti-OGT (1:1000, sc-74546, Santa Cruz Biotechnology, CA, USA), anti-GRP78 (1:1000, sc-13968, Santa Cruz Biotechnology, CA, USA), anti-GSK3β (1:1000, sc-377213, Santa Cruz Biotechnology, CA, USA), anti-p-tau (T231, 1:1000, ab194815), anti-Amyloid β (1:1000, NBP2-13075, Novos Biologicals), anti-CHOP (1:1000, sc-7351, Santa Cruz Biotechnology, CA, USA), anti-GRP94 (1:1000, sc-32249, Santa Cruz Biotechnology, CA, USA), anti-ATF-4 (1:1000, Cell Signaling Technology), anti-p-GSK3β (1:1000, 9336S, Cell Signaling Technology), anti-IBA-1 (1:1000, ab178846, Abcam, Cambridge, UK), GFAP (Sigma), anti-CD68 (1:1000, ab125212, Abcam, Cambridge, UK), IL-1β (1:1000, 12242 S, Cell Signaling Technology), TNF-a (1:1000, sc-52746, Santa Cruz Biotechnology), iNOS (1:1000, 610431, BD bioscience) and anti-β-actin (1:1000, sc-47778, Santa Cruz Biotechnology) overnight at 4 °C. The membranes were then incubated with horseradish peroxidase (HRP)-conjugated secondary antibodies and detected using an Enhanced Chemiluminescence (ECL) detection system (Promega). Densitometric quantification of protein bands was performed using the Bio-Rad Image Lab program (Bio-Rad).

### Thioflavin S staining

For thioflavin S staining, deparaffinized paraffin sections were incubated in a filtered 1% aqueous thioflavin S solution for 8 min at room temperature. Subsequently, the slides were washed in a series of ethanol (Duksan, Ansan, Korea) solutions in the order of 80%, 95%, and distilled water, with each wash step lasting 3 min. The slides were then examined under a confocal LSM 510 META microscope (Carl Zeiss) and analyzed using ImageJ software.

### Sholl analysis of microglia

Sholl analysis was performed to investigates the branching patterns of microglial processes, according to the previously described method [23]. In brief, the sections of the hippocampus were stained for IBA-1 and DAPI and captured as 63X z-stack images at 1 μm intervals using confocal LSM 510 META microscopy. A maximum intensity projection image of IBA-1 was created using ZEN Light software. To analyze intersections at each radius, the Sholl analysis plugin was employed, starting from the first shell at 50 μm and subsequent shells set at 2 μm step sizes. For quantification, intersections were evaluated to measure microglial ramification from the center of the cell soma to the furthest point. The “Analyze Skeletonize” plugin in ImageJ software was utilized to count the number of branches and branch lengths from each cell.

### Stereotaxic intracranial injection of AAV-OGT

To achieve the overexpression of mouse OGT, we utilized the pAAV-CMV-Myc-Flag vector for cloning. The viral vectors were pseudotyped, with the transgene of interest flanked by inverted terminal repeats of AAV8 and packaged in an AAV8 capsid. The mouse OGT vectors were purified, and virus production was carried out at the Korea Institute of Science and Technology (KIST) Virus Facility (KIST, Seoul, Korea). The mice were randomly assigned to one of three groups: vector-only (control), vector-only with CSD, and AAV-OGT with CSD. For the injection, mice were administered 4 µl of either the pAAV-CMV-Myc-Flag control virus or AAV8-OGT-Myc-Flag virus. Prior to the injection, the mice were anesthetized with isoflurane (Baxter, IL, USA). The viruses were then injected into the dentate gyrus (DG) of the hippocampus at specific coordinates (–2.1 mm posterior to bregma, ± 1.2 mm lateral to midline, and − 2.0 mm ventral to the brain surface) using a 26-gauge stainless steel injection needle connected to a 10 µl microsyringe (Hamilton) at a flow rate of 0.3 µl/min. After injection, the needle was kept in place for 10 min before being slowly withdrawn.

### Serum cortisol and corticosterone levels

Fresh blood samples were obtained from heart and then centrifuged at 2000 g at 4 °C for 20 min. The serum of cortisol (Mybiosource) and corticosterone (Abcam) were determined using enzyme linked immunosorbent assay (ELISA) kits, according to the manufacturer’s instructions.

### Statistical analysis

The data are presented as means ± SEM (standard error of the mean). Statistical analysis was conducted using GraphPad Prism 8 software. Data comparisons were performed using one-way analysis of variance (ANOVA), two-way ANOVA, or unpaired Student’s t-test, as appropriate. Differences were considered statistically significant at *p* < 0.05. Graphs were generated using GraphPad Prism 8.

## Results

### Cognitive dysfunction induced by CSD is improved by GlcN

**Fig. 1:**
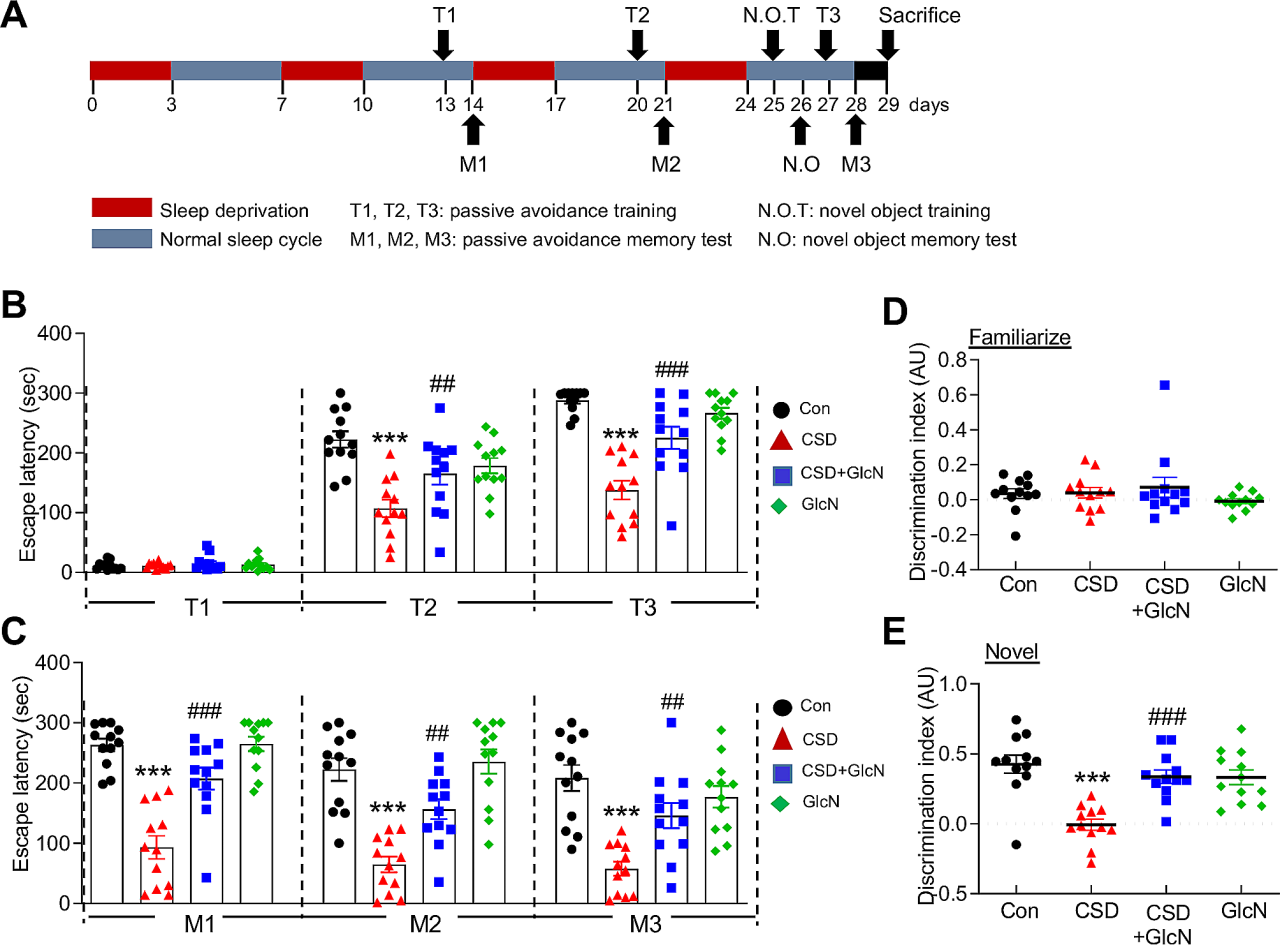
The effect of GlcN on L/M dysfunction in CSD mice. (**A**) Schematic experimental procedure of chronic REM sleep deprivation (CSD) timeline diagram. Mice were subjected to REM sleep deprivation for 3 consecutive days (red) using the modified multiple platform method, followed by a normal sleep-wake cycle for 4 days (blue). This pattern was repeated 4 times over a span of 28 days. (B and C) Mice were subjected to CSD, with or without GlcN. GlcN (200 mg/kg) was administered intraperitoneally three times a week during sleep deprivation cycle. (**B**) The bar graph represents the escape latency during learning trials of the first (T1), second (T2), and third (T3) passive avoidance tests at one-hour intervals following the first cycle of SD. (**C**) The bar graph represents escape latency in memory test performed after the second (M1), third (M2), and fourth (M3) cycle during CSD (*n* = 12/group). (E and F) The graphs represent the percentage of preference for novel and familiar objects in the Novel Object Recognition (NOR) test. In the familiar test session (**D**), mice were placed into a box containing two identical objects and allowed to explore the box for 10 min. The time spent exploring each object was recorded. In the novel test session (**E**), mice were placed into the box with one familiar object and one novel object, and the exploration time for each object was recorded during a 10-min period. The discrimination index (DI) was calculated using the formula DI = (exploration time of the novel object - exploration time of the familiar object) / (exploration time of the novel object + exploration time of the familiar object). The graph represents the DI in the control, GlcN, CSD, and CSD + GlcN groups (*n* = 12/group). Values are presented as mean ± SEM; ^***^p < 0.001 vs. control group; ^#^*p* < 0.05, ^##^*p* < 0.01, ^###^*p* < 0.001 vs. CSD group. Statistical analysis was performed using one-way ANOVA and two-way ANOVA with Tukey’s comparison; CSD, chronic sleep deprivation; Con, control; GlcN, glucosamine; T, learning trial; M, memory test

In this study, we sought to examine the impact of repeated and sustained chronic SD on the development of AD and explore the underlying mechanisms involved. We validated the SD model by confirming the increase in cortisol and corticosterone levels due to SD in the multi-platform SD model (supplementary Fig. 1). To this end, we generated a mouse model of chronic REM sleep deprivation (CSD) in which mice were subjected to SD for three consecutive days, followed by a 4-day rest period, a cycle that was repeated for a total of 4 weeks (28 days) (Fig. 1A). We investigated the alterations in brain *O*-GlcNAc cycling in response to CSD, and found that *O*-GlcNAcylation level was significantly reduced in the dentate gyrus (DG) and CA1 of hippocampus and cortex regions of the brains of CSD mice compared with control group mice (Supplementary Fig. 2A). Interestingly, the levels of OGA and OGT—enzymes involved in *O*-GlcNAcylation regulation—remained unchanged in the brains of CSD mice compared with mice in control groups (Supplementary Fig. 2B, 2C, 2D and 2E). To investigate the role of decreased *O*-GlcNAcylation in the development of CSD-induced brain dysfunction, we examined the effects of increasing *O*-GlcNAcylation through glucosamine (GlcN) supplementation. Specifically, we investigated the effects of daily GlcN administered throughout the duration of CSD on cognitive dysfunction, with a particular focus on L/M impairment induced by CSD. We performed two types of L/M tests. In the passive avoidance test, which measures fear memory, mice in the control group as well as GlcN-treated mice successfully learned to associate an electric shock with the dark room environment and retained this fear memory after 2, 3, and 4 weeks. However, CSD mice exhibited reduced learning ability and a lack of memory in this test (Supplementary Fig. 3, Fig. 1B and C). Importantly, intraperitoneal administration of GlcN to mice in the CSD group resulted in a notable restoration of L/M capability. In the NOR test, CSD mice exhibited a decline in their ability to recognize novel objects compared with control and GlcN-treated mice. However, mice in the GlcN-treated CSD group showed improvement in their ability to remember and recognize novel objects (Fig. 1D and E). Collectively, these findings indicate that GlcN effectively mitigates the long-term memory and fear memory dysfunction induced by CSD.

### Administration of GlcN rescues CSD-induced decreases in O-GlcNAcylation levels and dendritic spine density

**Fig. 2:**
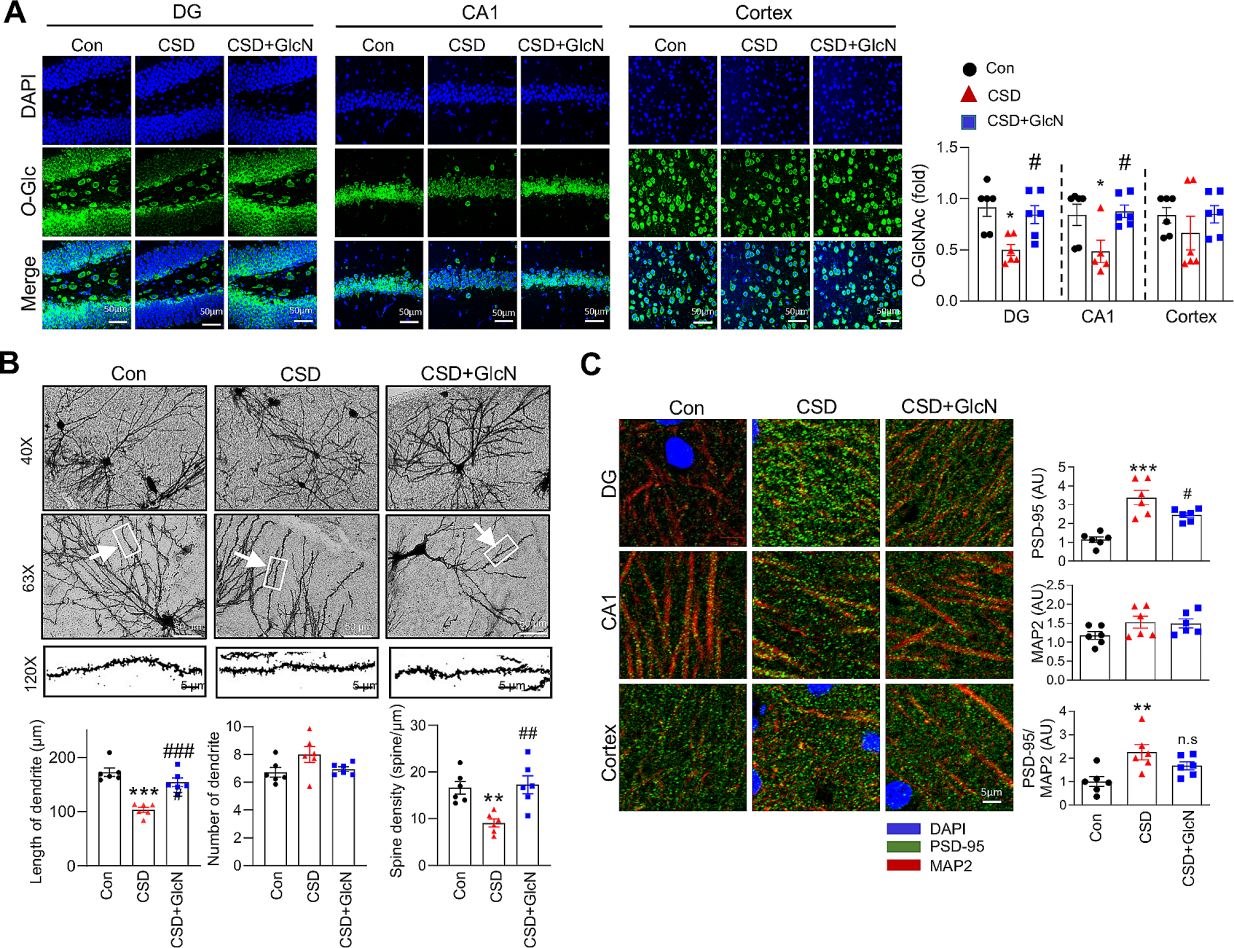
Effects of GlcN on *O*-GlcNAcylation and synaptic density in the brain of CSD Mice. (**A**) Representative confocal images (40X) of *O*-GlcNAc, DAPI, and merged images of the brain from control mice, and those subjected to CSD with or without GlcN. DAPI staining was performed for nuclei visualization. The scale bar represents 50 μm. The accompanying graph represents the quantification of *O*-GlcNAc levels in DA, CA1 and cortex of the brain (*n* = 6/group). (**B**) Mouse brains were stained using the Golgi-cox method. Slides were prepared from coronal sections with a thickness of 100 μm. Golgi-cox staining images of dendritic spines in the hippocampal CA1 region were captured at 40X, 63X, and 120X magnifications from control and CSD mice with or without GlcN. The length of each dendrite and the spine density were measured using Fiji Image J software. The scale bar represents 50 μm, and the scale bar for the enlarged images is 5 μm. The graphs present the quantification of dendrite length, number of dendrites, and spine density per µm (*n* = 6). (**C**) Staining images of PSD-95 (green), MAP-2 (red) and DAPI (blue) co-staining of DG, CA1 and cortex brain of control and CSD with or without GlcN mice (*n* = 6/group). The graphs represent the quantification of PSD-95, MAP2, and PSD-95/MAP2 ratio. The scale bar represents 5 μm. Values are presented as the mean ± SEM; ^*^p < 0.05, ^**^p < 0.01, ^***^p < 0.001, vs. control group; ^#^*p* < 0.05, ^###^*p* < 0.001 vs. CSD group. Statistical analysis was performed using one-way ANOVA with Tukey’s comparison. CSD, chronic sleep deprivation; Con, control; *O*-Glc, *O*-GlcNAc; DG, dentate gyrus; PSD-95, postsynaptic density-95; MAP2, microtubule-associated protein 2

A subsequent investigation of the effects of GlcN on CSD-induced *O*-GlcNAcylation levels showed that chronic GlcN treatment during CSD effectively restored *O*-GlcNAcylation to a level comparable to that in the control group in DG and CA1 regions of the mouse brain (Fig. 2A). We next assessed dendrite morphology and dendritic spine density, observing a reduction in dendrite length and dendritic spine density in the brains of CSD mice compared with control mice. Injection of GlcN reversed these reductions, indicating a beneficial effect of GlcN on dendrite morphology (Fig. 2B). In contrast, no changes in the number of dendrites were observed. (Fig. 2B). We further found that the levels of postsynaptic density protein-95 (PSD-95), as well as the ratio of PSD-95 to MAP-2 (microtubule-associated protein 2), were increased in the brains of CSD mice compared with those of control mice (Fig. 2C), suggesting that CSD alters synaptic organization. Interestingly, GlcN treatment effectively attenuated the CSD-induced increase in PSD-95 levels, suggesting its potential to mitigate the synaptic changes associated with CSD. However, the effect of GlcN on the PSD-95/MAP2 ratio was not as significant.

### GlcN attenuates the accumulation of Aβ and p-Tau induced by CSD while mitigating the CSD-induced decrease in O-GlcNAc-Tau levels

**Fig. 3:**
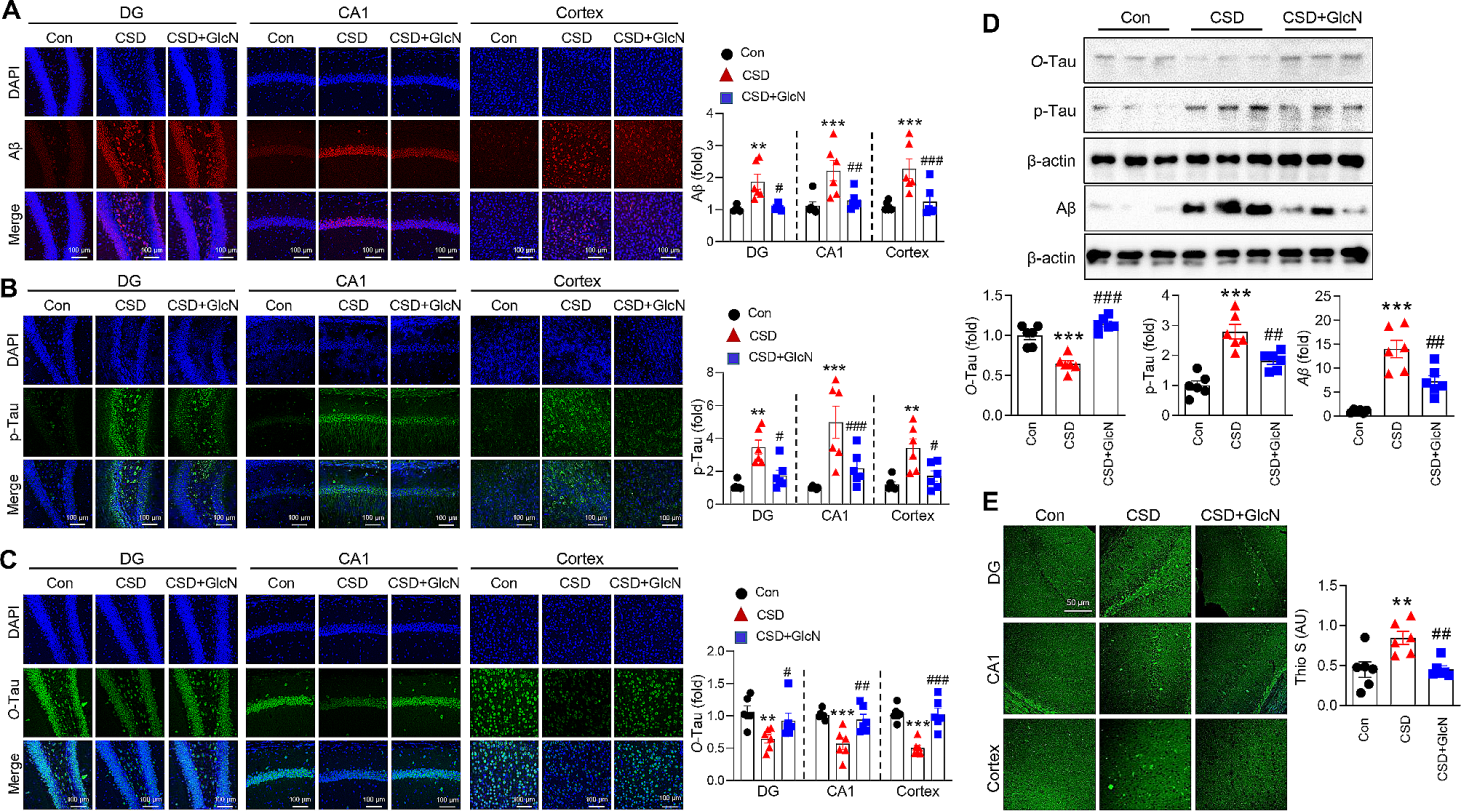
The effect of GlcN on CSD-induced Aβ, p-Tau and*O*-Tau accumulation in the brain of mice. (**A-C**) Representative confocal images of Aβ (20X) (**A**), p-Tau (20X) (**B**), or *O*-Tau (20X) (**C**), as well as DAPI and merged images of the brain from control mice and mice subjected to CSD with or without GlcN. The graphs represent the quantification of Aβ (*n* = 6/group), p-Tau and *O*-Tau in the mouse brain (*n* = 6/group). Values are presented as the mean ± SEM. (**D**) Western blot analysis using antibodies specific for O-tau, p-tau, Aβ and β-actin was performed on the hippocampus of control and CSD mice with or without GlcN. The graphs represent the densitometric quantification of O-tau, p-tau, and Aβ normalized by β-actin (*n* = 6/group). (**E**) Representative images of thioflavin S staining are shown, and the graph presents the quantification of thioflavin S staining (*n* = 6/group). The scale bar represents 50 μm. Values are measured as the mean ± SEM. Statistical analysis was performed using one-way ANOVA with Tukey’s post-hoc comparison; ^**^p < 0.01, ^***^p < 0.001, vs. control group; ^#^*p* < 0.05, ^##^*p* < 0.01, ^###^*p* < 0.001 vs. CSD group; CSD, chronic sleep deprivation; Con, control; DG, dentate gyrus; *O*-Tau, GlcNAc-Tau; Thio, thioflavin S; Aβ, amyloid beta

Assessments of AD pathology in the brains of mice injected daily with GlcN during CSD revealed that GlcN effectively decreased Aβ accumulation induced by CSD (Fig. 3A and D). Neurofilamentary tangles in AD also contain abnormal p-Tau. In addition, Tau is subjected to *O*-GlcNAcylation, which inversely modulates Tau phosphorylation [24]. Therefore, we compared the levels of Tau phosphorylation and *O*-GlcNAcylation in response to CSD, with or without GlcN treatment. This analysis showed that CSD significantly increased the level of p-Tau in the brain, and that this increase was suppressed by GlcN (Fig. 3B and D). Conversely, *O*-GlcNAcylated-Tau (*O*-Tau) was decreased by CSD. Again, this effect was reverse by treatment with GlcN, which restored *O*-Tau to levels similar to those in the control group (Fig. 3C and D). Thioflavin S staining further demonstrated that CSD increased protein aggregates containing Aβ and showed that this increase was inhibited by GlcN treatment (Fig. 3E).

### GlcN treatment effectively inhibits CSD-induced glial activation

**Fig. 4:**
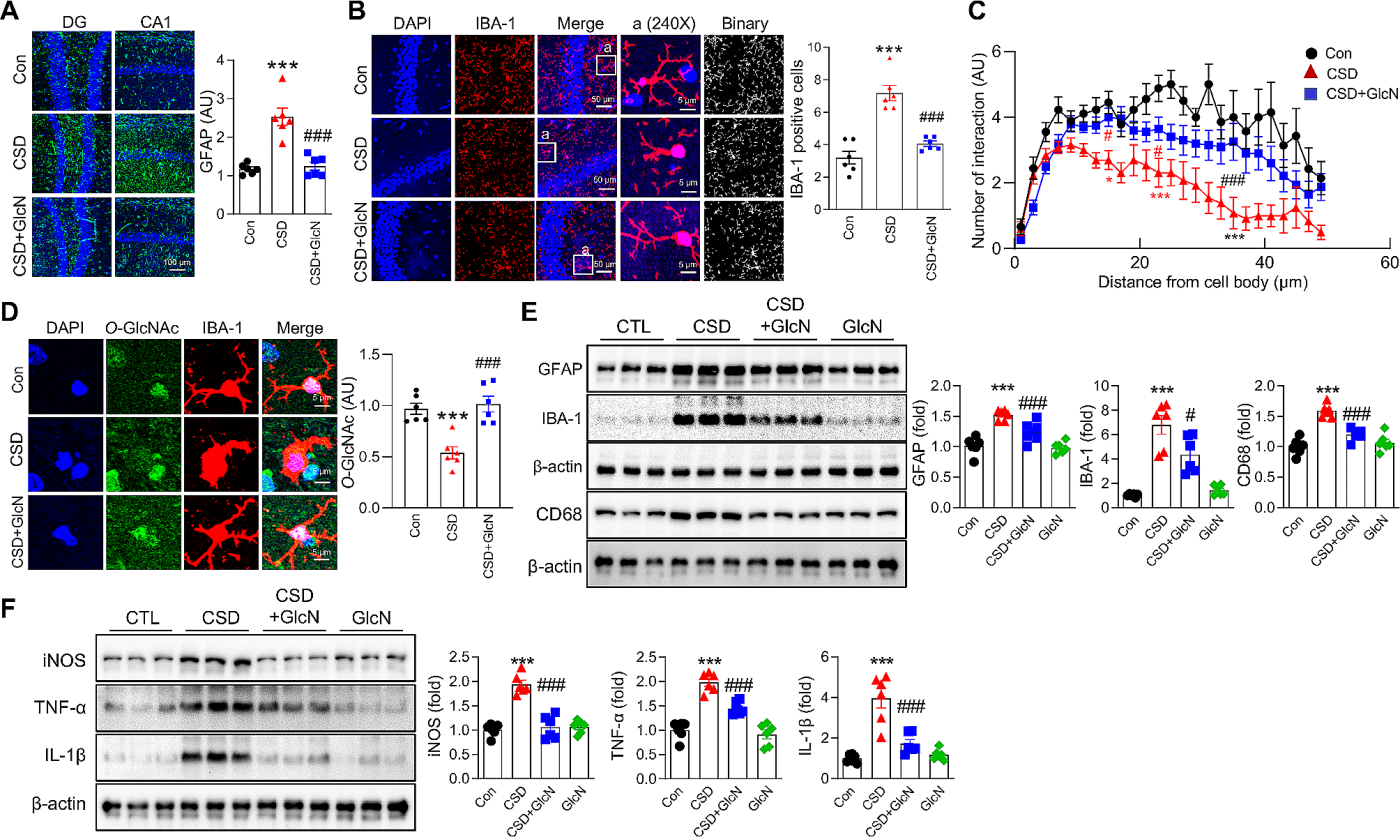
Neuroinflammation induced by CSD with or without GlcN in the mouse brain. (**A**) Representative immunofluorescent staining of GFAP (an activated astrocyte marker), DAPI, and merged images of the brain from control mice, and mice subjected to CSD with or without GlcN, are shown. The scale bar represents 100 μm. The graph represents the densitometric quantification of GFAP (*n* = 6 per group). (**B**) Representative confocal images (40X and 240X) of IBA-1, DAPI, and merged images, as well as binary images for Sholl analysis, from the mice brain samples of control and CSD groups, with or without GlcN (*n* = 6/group). Enlarged images are presented in the white boxes. The scale bar is set at 50 μm for the main images and 5 μm for the enlarged images. (**C**) The graph represents the intersections from the center of the cell soma to the furthest point of the microglial Sholl image in the CA1 region of the mice brain, using ZEN Light software (*n* = 9 ~ 20 cells/group). The Sholl analysis plugin was utilized, with the first shell set at 50 μm and subsequent shells set at 2 μm step sizes. (**D**) Z-stack staining images of IBA-1 (red), DAPI (blue), and *O*-GlcNAc (green), along with merged images from the brains of control and CSD mice with or without GlcN. The scale bar is set at 5 μm. The graph represents the quantification of *O*-GlcNAc levels within IBA-1 positive cells (*n* = 6/group). (**E**) Western blot analysis using antibodies specific for GFAP, IBA-1, CD68, and β-actin was performed on the hippocampus of control and CSD mice with or without GlcN. The graphs represent the densitometric quantification of GFAP, IBA-1, and CD68 normalized by β-actin (*n* = 6/group). (**F**) Western blot analysis using antibodies specific for iNOS, TNF-α, IL-1β, and β-actin was performed on the hippocampus of control and CSD mice with or without GlcN. The graphs represent the densitometric quantification of iNOS, TNF-α, and IL-1β normalized by β-actin (*n* = 6/group). Values are presented as mean ± SEM; ^*^p < 0.05, ^**^p < 0.01, ^***^p < 0.001 vs. control group; ^#^*p* < 0.05, ^##^*p* < 0.01, ^###^*p* < 0.001 vs. CSD group; (**A**), (**B**), (**D**), (**E**), and (**F**) were analyzed using one-way ANOVA with Tukey’s post-hoc comparison. (**C**) was analyzed using two-way ANOVA with Tukey’s post-hoc comparison. CSD, chronic sleep deprivation; Con, control; DG, dentate gyrus; GFAP, glial fibrillary acidic protein; IBA-1, ionized calcium-binding adapter molecule 1; TNF-α, tumor necrosis factor-α; IL-1β, interleukin-1β; iNOS, inducible nitric oxide synthase

We next investigated the effects of CSD on glial activation in the brain and further compared the effects of GlcN in modulating these responses. We found that CSD led to an increase in astrocyte activation, as indicated by elevated levels of glial fibrillary acidic protein (GFAP) (Fig. 4A). Additionally, the number of IBA1-positive (activated) microglia in the brains of CSD mice was increased (Fig. 4B). Both the increase in GFAP and the presence of IBA1-positive cells were significantly reduced by GlcN treatment. Furthermore, a Sholl analysis of IBA1-positive cells revealed a decrease in the number of Sholl intersections in microglia from CSD mouse brains compared with microglia from control mouse brains. CSD mice treated with GlcN exhibited an increase in the number of Sholl intersections compared with mice in the CSD group (Fig. 4C). A subsequent examination of changes in *O*-GlcNAcylation in microglia in response to CSD showed that CSD significantly reduced *O*-GlcNAc levels in activated microglia (Fig. 4D), an effect that was reversed by administration of GlcN (Fig. 4D). Immunoblot analyses, performed to further investigate glial activation and inflammatory cytokines, revealed an increase in GFAP, IBA-1, CD68, iNOS, TNF-α, and IL-1β levels in the CSD group, and further showed that treatment with GlcN significantly diminished the expression of these proteins (Fig. 4E and F).

### GlcN inhibits CSD-induced stimulation of ER stress and activation of GSK3β in the brain

**Fig. 5:**
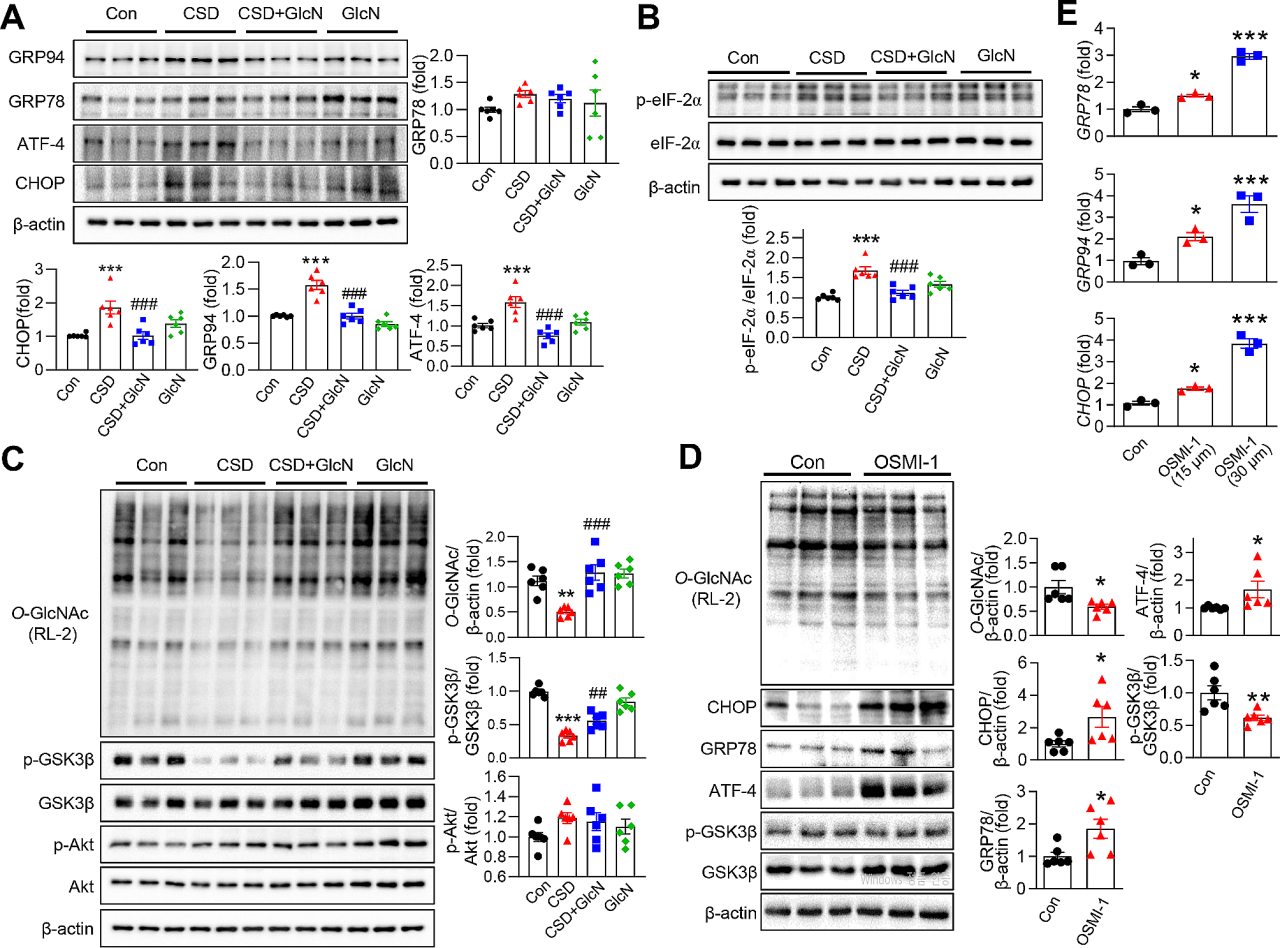
The effect of CSD or OSMI-1 on ER Stress and GSK3β activation in the mouse brain and N2a cells. (**A**) Representative Western blot images of GRP78, GRP94, CHOP, ATF-4, and β-actin from the hippocampus of mice brains. The graphs represent the densitometric quantification normalized by β-actin as a control (*n* = 6/group). (**B**) Representative Western blot images of p-eIF2α, eIF2α, and β-actin from the hippocampus of mice brains. The graph illustrates the densitometric quantification of p-eIF2α normalized to eIF2α as a control (*n* = 6/group). (**C**) Representative Western blot images of *O*-GlcNAc (RL-2), p-GSK3β, GSK3β, and β-actin from the hippocampus of mice brains. The graphs represent the densitometric quantification of *O*-GlcNAc normalized to β-actin and p-GSK3β normalized to GSK3β (*n* = 6/group). (**D**) Neuro2a cells were treated with 30 µM OSMI-1 for 24 h. Whole-cell lysates were subjected to immunoblotting with antibodies against RL-2 (*O*-GlcNAc), CHOP, GRP78, ATF-4, p-GSK3β, GSK3β, and β-actin. The relative densitometric intensities of *O*-GlcNAc, CHOP, GRP78, and ATF-4 were quantified by normalizing to β-actin. The intensity of p-GSK3β was measured by normalizing to GSK3β. (**E**) Neuro2a cells were treated with either 15 µM or 30 µM OSMI-1 for 24 h. Quantification of GRP78, GRP94, and CHOP mRNA levels was performed using quantitative real-time PCR. The values are presented as mean ± SEM.; ^*^p < 0.05, ^**^p < 0.01, ^***^p < 0.001 vs. control group; ^##^*p* < 0.01, ^###^*p* < 0.001 vs. CSD group. Statistical analysis for (**A**), (**B**), and (**C**) was performed using One-way ANOVA with Tukey’s post-hoc comparison. Statistical analysis for (**D**) and (**E**) was conducted using Student’s t-test. CSD, chronic sleep deprivation; Con, control; GSK3 β, glycogen synthase kinase 3 beta; Akt, protein kinase B; GRP, glucose-regulated protein; CHOP, C/EBP homologous protein; eIF2α, eukaryotic translation initiation factor 2 A; ATF-4, activating transcription factor 4; GRP94, glucose-regulated protein 94

Several previous studies have demonstrated an association between ER stress and neurodegenerative diseases, including AD. To investigate whether CSD induces ER stress, we examined the expression levels of ER stress-related proteins in the hippocampus. This analysis revealed a significant increase in the expression of GRP78, GRP94, CHOP, and ATF-4 in the brains of CSD mice; it also showed that this increase was effectively suppressed by GlcN treatment (Fig. 5A). CSD was further found to activate eukaryotic translation initiation factor 2 (eIF2α), an effect that was also inhibited by GlcN treatment (Fig. 5B). Next, we investigated CSD-induced changes in GSK3β signaling. This pathway was of interest because it has been proposed that GSK3β is not only a downstream target of ER stress, but also a kinase involved in Tau phosphorylation and Aβ processing [25, 26]. The brains of mice from the CSD group exhibited a reduction in GSK3β phosphorylation at serine 9 indicative of activation (Fig. 5C). Moreover, GlcN treatment effectively suppressed this CSD-induced effect on GSK3β activation. To investigate the potential involvement of *O*-GlcNAc cycling in ER stress and GSK3β activation, we examined the effect of the OGT inhibitor, OSMI-1, in N2a cells. These experiments demonstrated that OSMI-1 treatment led to a decrease in *O*-GlcNAcylation, indicating inhibition of OGT activity. OSMI-1 treatment resulted in increased expression of the ER stress markers, CHOP, GRP78, and ATF-4; it also increased phosphorylation of GSK3β (Fig. 5D). Consistent with this, mRNA levels of CHOP, GRP78, and ATF-4 were increased in a dose-dependent manner by OSMI-1, providing further evidence that OSMI-1 induces ER stress (Fig. 5E).

### Minocycline suppresses CSD-induced glial activation and AD pathogenesis and rescues the CSD-induced decrease in O-GlcNAcylation in the brain

**Fig. 6:**
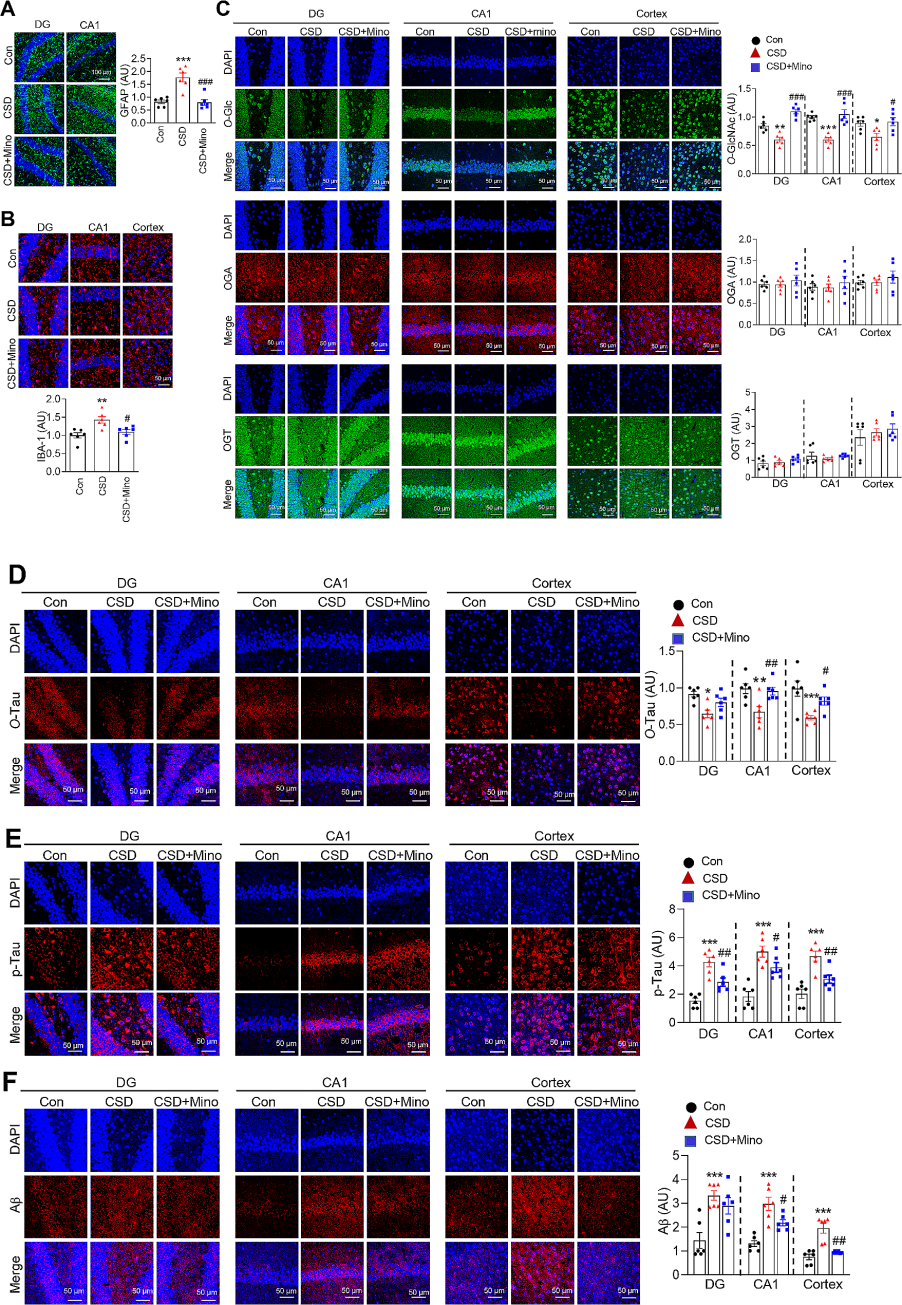
Effects of minocycline on neuroinflammation, *O*-GlcNAcylation, and degenerative changes in the brain induced by CSD. During CSD, the mice were administered minocycline (10 mg/kg) three times per week. (**A**) Representative immunofluorescent staining images (20X) of GFAP, DAPI, and merged images of the brain from the control, CSD, and CSD plus minocycline groups. The scale bar represents 100 μm. The graph represents the densitometric quantification of GFAP levels (*n* = 6/groups). (**B**) Representative immunofluorescent staining images (40X) of IBA-1, DAPI, and merged images for the control, CSD, and CSD plus minocycline groups (*n* = 6 per group). The graph illustrates the densitometric quantification of IBA-1 levels. A scale bar of 50 μm is included in the images. (**C**) Representative confocal staining images (40X) of *O*-GlcNAc, OGA, OGT, DAPI, and merged images of the mouse brain from the control, CSD, and CSD plus minocycline group. The scale bar represents 50 μm. Quantification of *O*-GlcNAc, OGA, and OGT levels in the brain was conducted (*n* = 6/group). (**D** ~ **F**) Representative confocal images (40X) of O-Tau (**D**), p-Tau (**E**), Aβ (**F**), along with DAPI and merged images of mouse brains from the control, CSD, and CSD plus minocycline groups. A scale bar of 50 μm is provided. The graphs depict the quantification of *O*-Tau (**D**), p-Tau (**E**), and Aβ (**F**) levels (*n* = 6/group). The values are expressed as the mean ± SEM; ^*^p < 0.05, ^**^p < 0.01, ^***^p < 0.001 vs. control group; ^#^*p* < 0.05, ^##^*p* < 0.01, ^###^*p* < 0.001 vs. CSD group. Statistical analysis was performed using One-way ANOVA with Tukey’s comparison. CSD, chronic sleep deprivation; Con, control; mino, minocycline; *O*-Glc, *O*-GlcNAc; OGA, *O*-GlcNAcase; OGT, *O*-GlcNAc transferase; GFAP, glial fibrillary acidic protein; IBA-1, ionized calcium-binding adapter molecule 1; *O*-Tau, *O*-GlcNAc-Tau; p-Tau, phosphorylated-Tau; Aβ, amyloid beta

We further investigated the impact of minocycline, a well-known anti-inflammatory drug, on CSD-induced brain pathogenesis and *O*-GlcNAc cycling. We observed that daily administration of minocycline during CSD effectively suppressed CSD-induced activation of astrocytes and microglia, as evidenced by decreased levels of GFAP and IBA-1, respectively (Fig. 6A and B). A subsequent examination of the effects of minocycline on CSD-induced *O*-GlcNAc levels and the expression of OGT and OGA revealed a significant reversal of the CSD-induced *O*-GlcNAcylation reduction in the mouse brain (Fig. 6C). The expression of OGT and OGA did not change significantly following CSD induction and was unaffected by minocycline (Fig. 6C). We further observed that minocycline mitigated the CSD-induced increase in p-Tau levels and reversed the CSD-induced decrease in *O*-Tau levels (Fig. 6D and E). Moreover, minocycline effectively reduced the CSD-induced accumulation of Aβ (Fig. 6F). Experiments testing the influence of minocycline on CSD-induced ER stress and GSK3β activation showed no significant effects (supplementary Fig. 4).

### Hippocampal overexpression of OGT restores CSD-induced L/M dysfunction

**Fig. 7:**
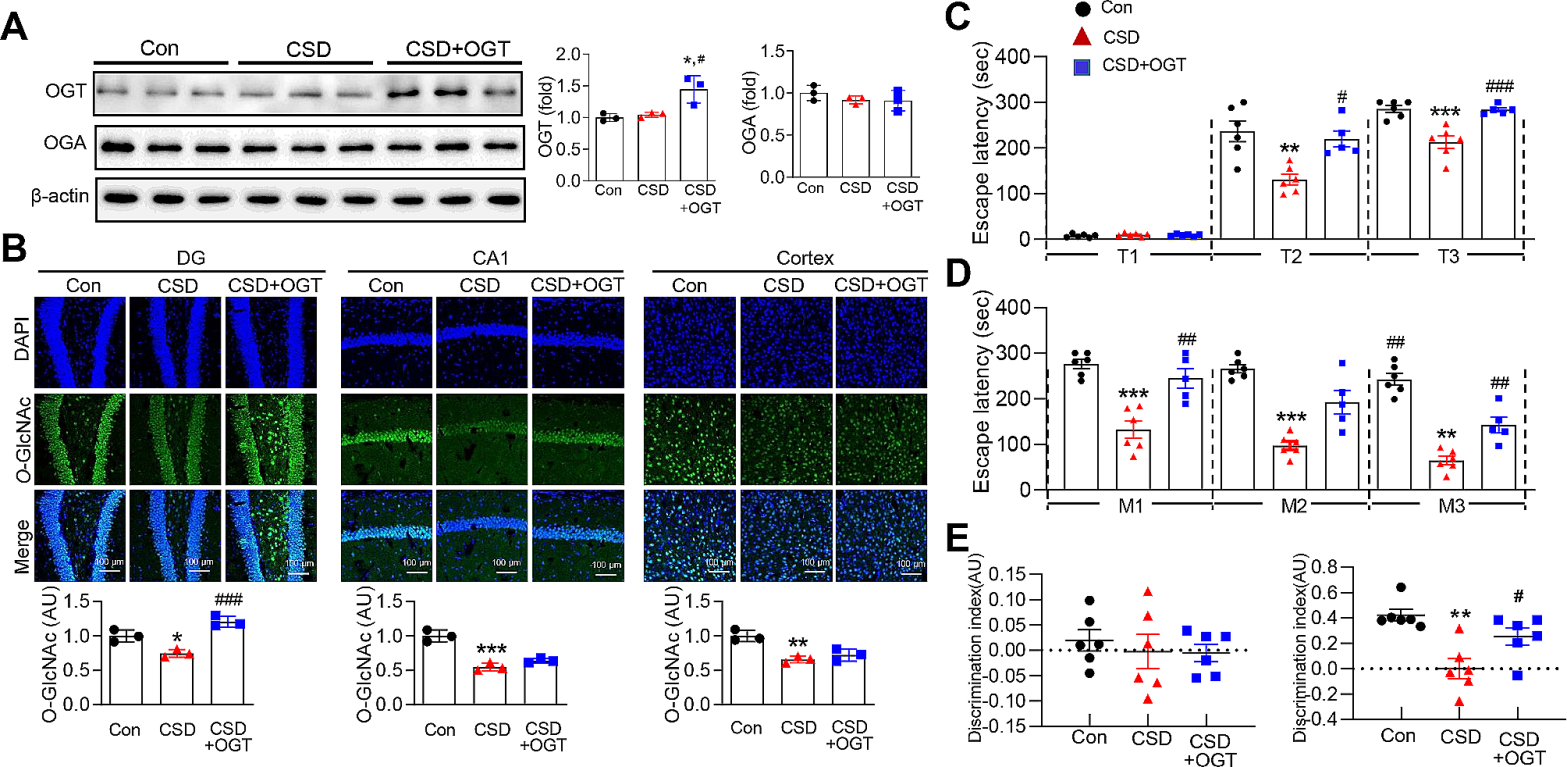
The Effect of OGT overexpression on CSD-induced L/M dysfunction. AAV-OGT or control viruses were injected into the DG of mice brains, and then the mice were subjected to SD for four cycles to induce CSD. (**A**) Representative western blot analysis and quantification of OGT and OGA expression levels in the hippocampus from the control, CSD, and CSD plus OGT group. (**B**) Representative confocal image (20X) of *O*-GlcNAc, DAPI and merged immunofluorescence staining in the DG, CA1, and cortex of the mouse brain. Graphs represent densitometric quantification of *O*-GlcNAc (*n* = 3/group). The scale bar represents 100 μm. (**C**) The graph displays the escape latency during the learning trials of the first (T1), second (T2), and third (T3) passive avoidance tests at one-hour intervals following the first cycle of SD (*n* = 6/group). (**D**) The graph represents the escape latency in the memory test conducted after the second (M1), third (M2), and fourth (M3) cycle during CSD (*n* = 6/group). (**E**) The NOR test was conducted to measure the percentage of preference for familiar and novel objects. In the familiar test session (left), mice were placed into a box containing two identical objects and the time spent exploring each object was recorded. In the novel test session (right), mice were placed into the box with one familiar object and one novel object, and the exploration time for each object was recorded. The discrimination index (DI) was calculated using the formula DI = (exploration time of the novel object - exploration time of the familiar object) / (exploration time of the novel object + exploration time of the familiar object). The graph represents the DI in the control, CSD, and CSD + OGT groups (*n* = 6/group). The values are expressed as the mean ± SEM; ^**^p < 0.01, ^*^p < 0.01, ^***^p < 0.001 vs. control group; ^#^*p* < 0.05, ^##^*p* < 0.01, ^###^*p* < 0.001 vs. CSD group. Statistical analysis was performed using One-way ANOVA with Tukey’s comparison. CSD, chronic sleep deprivation; *O*-Glc, *O*-GlcNAc; OGA, *O*-GlcNAcase; OGT, *O*-GlcNAc transferase; DG, dentate gyrus; T, learning trial; M, memory test

To further elucidate the role of alterations in *O*-GlcNAc cycling in CSD-induced brain pathogenesis, we utilized a viral delivery system, employing an AAV vector to overexpress OGT in the hippocampus. Injection of OGT viruses into the hippocampal DG of CSD mice induced a significant increase in OGT expression (Fig. 7A). Consistent with this, *O*-GlcNAc levels were increased specifically within the DG of CSD mice administered OGT viruses compared with uninjected CSD mice. No significant differences in *O*-GlcNAc levels were observe in the CA1 region or cortex, confirming the selective targeting of the DG region (Fig. 7B). We then assessed the impact of OGT overexpression on L/M functions. In the passive avoidance test, which measures fear memory, OGT overexpression in CSD mice significant restored L/M functions compared with CSD mice that did not receive viral injections (Fig. 7C and D). In the NOR test, mice with DG-specific OGT overexpression exhibited an enhanced ability to remember and willingness to explore novel objects (Fig. 7E).

### OGT overexpression alleviates CSD-induced ER stress and AD pathology

**Fig. 8:**
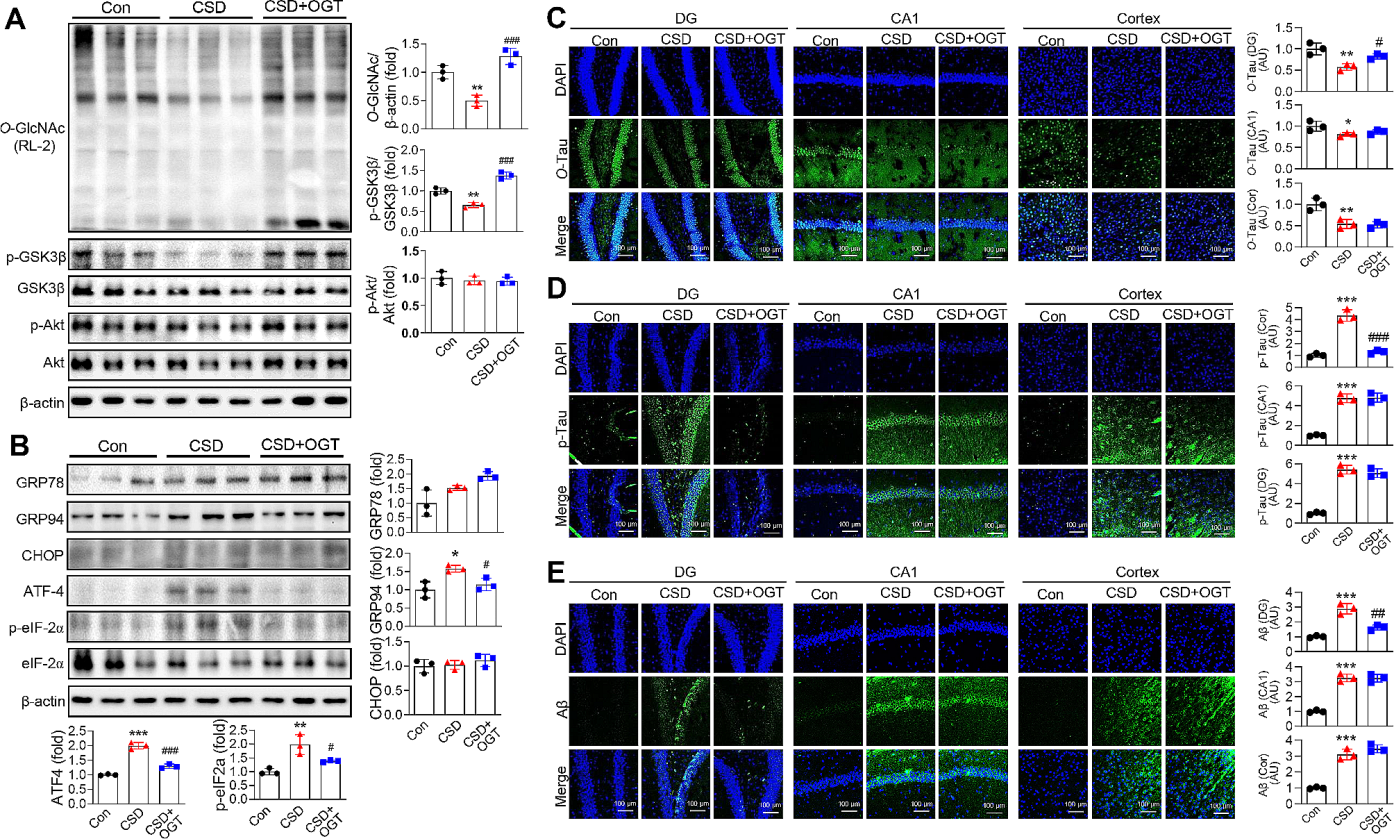
Effects of OGT Overexpression on ER Stress induction and accumulation of p-Tau, *O*-Tau, and Aβ induced by CSD. AAV-OGT or control viruses were injected into the DG of mice brains, and then the mice were subjected to SD for four cycles to induce RSD. (**A**) Representative western blot analysis of *O*-GlcNAc and p-GSK3β, in hippocampus from the control, CSD, and CSD plus OGT group. The graphs represent the densitometric quantification of *O*-GlcNAc levels normalized to β-actin and p-GSK3β levels normalized to GSK3β (*n* = 3/group). (**B**) Representative western blot analysis for GRP78, GRP94, CHOP, ATF-4, p-eIF2α and eIF2α. The graphs represent densitometric quantification of Western blots, normalized to β-actin (*n* = 3/group). (**C-E**) Representative confocal images (20X) of *O*-Tau (**C**), p-Tau (**D**), Aβ (**E**) and merged immunofluorescence staining in the DG, CA1, and cortex of mice brain. Nuclei were counterstained with DAPI. The scale bar represents 100 μm. The values are expressed as the mean ± SEM; ^**^p < 0.01, ^***^p < 0.001 vs. control group; ^#^*p* < 0.05, ^##^*p* < 0.01, ^###^*p* < 0.001 vs. CSD group. Statistical analysis was performed using One-way ANOVA with Tukey’s comparison. CSD, chronic sleep deprivation; Con, control; DG, dentate gyrus; *O*-Tau, *O*-GlcNAc-Tau; p-Tau, phosphorylated-Tau; Aβ, amyloid beta; GSK3β, glycogen synthase kinase 3 beta; GRP78, glucose-regulated protein78; CHOP, C/EBP homologous protein; ATF-4, activating transcription factor 4; GRP94, glucose-regulated protein 94; eIF2α, eukaryotic translation initiation factor 2 A

CSD mice that received OGT virus injections exhibited increased levels of *O*-GlcNAc and p-GSK3β compared with uninjected CSD mice (Fig. 8A). They also showed significant elevations in protein levels of the ER stress markers, GRP94, ATF4, and p-eIF2α. Notably, this increase was reversed by OGT overexpression in the DG, which restored these proteins to levels comparable to those in normal controls (Fig. 8B). We further found that the decrease in *O*-Tau levels observed in CSD mice was specifically reversed by overexpression of OGT in the DG region of the brain (Fig. 8C). Conversely, p-Tau levels, which were increased in response to CSD, were reduced by OGT overexpression in the DG region of the CSD mouse brain (Fig. 8D). OGT overexpression also suppressed the CSD-induced accumulation of Aβ in the DG region. Importantly, the CSD-induced changes in *O*-Tau, p-Tau, and Aβ levels were not affected by OGT overexpression in the CA1 region or cortex of CSD mice (Fig. 8D and E).

## Discussion

Growing evidence has demonstrated that CSD induces cognitive impairment, which is also a clinical characteristic of AD, and worsens the pathological changes associated with AD [27, 28]. The impact of SD on neurocognitive functions may be attributable, at least in part, to disruptions in cerebral glucose metabolism. Deep sleep is characterized by minimal glucose demand in the brain, whereas during REM sleep, the level of metabolism is comparable to that in wakefulness [29]. However, the importance of cerebral glucose metabolism pathways during REMSD and the mechanisms that regulate them have historically been relatively understudied. In our previous research, we reported compelling evidence suggesting that dynamic changes in the cerebral glucose metabolism of the HBP in the brain may play a critical role in the cognitive impairment associated with SD [16, 17]. Building upon previous research, the present study aimed to investigate the hypothesis that repetitive and prolonged REMSD would lead to recurrent disturbances in cerebral HBP flux and subsequently affect *O*-GlcNAc cycling, thereby contributing to the pathogenesis of AD. Our study revealed a significant decrease in *O*-GlcNAcylation in the brains of CSD mice compared with normal control mice. Interestingly, however, unlike the case of a single episode of SD, where OGT decreases and OGA increases [17], CSD had no effect on OGA or OGT levels. This indicates that repetitive SD and subsequent recovery may contribute to dynamic reciprocal regulation of OGT and OGA expression, potentially leading to compensatory mechanisms between them. As a result, although the overall levels of *O*-GlcNAc may decrease, the intricate balance between OGT and OGA expression is preserved. Similarly, a previous report demonstrated that OGA and OGT levels remain unchanged while *O*-GlcNAcylation levels are decreased in the brains of 5xFAD and 3xTg AD model mice [30].

Sleep plays a role in dendritic spine formation and elimination [31, 32]. Specially, REM sleep is required for strengthening the new spine for long-term memory formation [33]. In our previous work, we found that a single episode of REMSD induces a decrease in dendrite length and number and reduces spine density [17]. Similarly, we found that CSD reduces dendrite length and spine density, both of which are reversed by GlcN treatment. In contrast, there was no change in the number of dendrites. Typically, SD has been reported to decrease both the number and density of dendritic spines, but it also has reported that, during REM sleep, the elimination of dendritic spines to strengthen new spines facilitates long-term potentiation [34]. Therefore, it is likely that the extent and type of SD can have diverse effects on dendritic spine number. Nonetheless, it is evident that the decrease in dendritic spine density in CSD mice points to diminished synaptic density, implying weakened neural connectivity and reduced information transmission, with consequent impacts on the development of cognitive disabilities.

The accumulation of extracellular Aβ plaques and increased intraneuronal Tau expression are significant pathological events in AD [35, 36]. Studies in transgenic mouse models of AD have also demonstrated an increase in Aβ plaques in the hippocampus following CSD [37]. In this study, we employed a 28-day repeated SD, demonstrating that this CSD paradigm led to increased deposition of Aβ and p-Tau, accompanied by reduced levels of *O*GlcNAcylation in the hippocampus and cortex regions of wild-type C57 BL/6 mice. To investigate the potential significance of decreased brain *O*-GlcNAc in CSD-induced AD pathology, we examined the effects of GlcN, which can serve as a direct substrate of *O*-GlcNAc, on these processes. Our findings strongly indicate that the restoration of *O*-GlcNAc by GlcN significantly contributes to the overall recovery from CSD-induced impairments in L/M, synaptic plasticity, and AD pathology. By overexpressing OGT in the mouse brain using the AAV system, we obtained additional strong confirmation that increasing *O*-GlcNAc levels can restore various neuropathogenic processes impaired by CSD. These findings confirm the crucial role of *O*-GlcNAc cycling in CSD-induced AD pathogenesis. Given that multiple lines of evidence demonstrate that *O*-GlcNAcylation limits Tau phosphorylation and aggregation [38–42], it is plausible that CSD might increase Tau phosphorylation through decreased *O*-GlcNAc cycling. Consistent with this, our results indicate that CSD leads to a decrease in brain expression levels of *O*GlcNAcylated-Tau (*O*-Tau) but conversely causes an increase in p-Tau levels. Moreover, treatment with GlcN reversed both of these effects, reducing p-Tau levels while simultaneously promoting an increase in *O*-Tau levels.

There is ongoing controversy regarding the role of *O*-GlcNAc cycling in regulating cognitive function and the development of AD. This controversy arises from reports of both increased and decreased *O*-GlcNAc levels in the AD brain [40, 43–47]. The divergent roles of *O*-GlcNAc in AD can be attributed to several factors, including variations in animal models, types of stimuli, and fluctuations in *O*-GlcNAc regulatory elements. However, we propose that maintaining the dynamic equilibrium of normal *O*-GlcNAc cycling in the brain is paramount to ensuring optimal brain function. For instance, we found that, whereas GlcN was capable of reversing the AD pathogenesis induced by CSD, we have also discovered that higher (10-fold) concentrations of GlcN alone lead to notable cognitive impairments in zebrafish and mouse models (unpublished observation), highlighting the importance of maintaining *O*GlcNAcylation homeostasis. Additionally, studies have demonstrated a robust 24-hour rhythm of *O*-GlcNAcylation in Drosophila, regulated by the expression and activity of OGT and OGA [48]. This rhythm is influenced by the integration of circadian and metabolic signals, further emphasizing the significance of sustaining the dynamic equilibrium of *O*-GlcNAc cycling. However, one of the important issues regarding the CSD-induced *O*-GlcNAcylation changes and subsequent cognitive impairment is the potential involvement of the stress response. The interplay between sleep deprivation and stress has been well-documented, with sleep deprivation being known to exacerbate stress [49]. Stress can increase insulin and glucose levels, leading to metabolic changes that are also observed under CSD conditions. In our study, it remains challenging to clearly distinguish whether the changes in *O*-GlcNAc are solely due to CSD or if they are significantly influenced by the stress response triggered by CSD.

Emerging evidence indicates that SD leads to cognitive impairment, primarily by inducing neuroinflammation, which disrupts hippocampal neuronal plasticity and impairs memory processes [50]. Furthermore, neuroinflammation plays a pivotal role in the development of SD-associated AD, because it initiates and exacerbates the accumulation of Aβ and p-Tau proteins [2, 51, 52]. Glial cells, including microglia and astrocytes, are densely located in the hippocampus and constitute the major components of the neuroimmune system [53]. In this study, we observed that CSD-induced activation of astrocytes and microglia was effectively suppressed by GlcN administration or OGT overexpression, indicating the potential importance of *O*-GlcNAc cycling in regulating neuroinflammation. We further observed a reduction in *O*-GlcNAcylation in microglia from CSD mice, a result consistent with previous findings showing decreased *O*-GlcNAcylation in activated microglia following ischemic brain injury or LPS stimulation [54, 55]. We previously demonstrated that activation of glial cells by similar insults (i.e., brain ischemia/reperfusion injury and hypoxic brain damage) is effectively inhibited by GlcN-mediated regulation of *O*-GlcNAc [55, 56]. Therefore, the demonstration here that GlcN or OGT overexpression suppresses neuroinflammation holds promise for mitigating the cognitive impairment and AD pathology induced by CSD. However, whether *O*-GlcNAc cycling directly inhibits glial activation or regulates inflammation through the modulation of other neural cell activities remains uncertain. As part of our exploration of the relationship between neuroinflammation and *O*-GlcNAc cycling, we assessed the effects of minocycline—an antibacterial agent known for its anti-inflammatory properties in the central nervous system [57, 58]—on CSD-induced brain dysfunction and *O*-GlcNAcylation. We found that minocycline-induced suppression of glial activation not only led to a decrease in CSD-induced p-Tau and Aβ accumulation, it also rescued the CSD-induced decrease in *O*-GlcNAcylation levels in the brain, restoring them to a level similar to that in the normal state. These results suggest the possibility that activated glial cells or inflammatory factors directly contribute to the regulation of *O*-GlcNAc levels in the brain. However, it is also plausible that the inflammatory response affects overall brain metabolism and neuronal function, thereby modulating *O*-GlcNAc.

Research from a different perspective has suggested that ER stress plays a critical role in the onset and progression of AD [59]; consistent with this, ER stress features prominently in the brains of AD patients [60]. One emerging link between ER stress and AD is glycogen synthase kinase-3 (GSK‐3) signaling. Studies have shown that increased GSK3 activity induces memory deficits [61, 62] and is a prominent feature of the AD brain [63]. The identified functions of GSK3 in the brain center on effects on Tau phosphorylation at most AD sites [64, 65], and Aβ overproduction [66]. In the current study, we demonstrated that the ER stress markers were increased in the brains of CSD mice, whereas phosphorylation of GSK3β was decreased, indicating its activation. Given that GlcN and OGT overexpression shared the property of reversing these molecular changes, we hypothesized that *O*-GlcNAc cycling might regulate ER stress and/or GSK3 signaling. Although previous studies have demonstrated activation of GSK-3β during ER stress [67, 68], the nature of the interplay between ER stress and GSK-3β signaling in AD has not yet been fully elucidated. In this study, the OGT inhibitor, OSMI-1, increased ER stress and GSK‐3β activation in N2a cells, indicating that *O*-GlcNAc cycling plays an important role in the regulation of both ER stress and GSK‐3β signaling. Consistent with this, it has been reported that increasing *O*-GlcNAcylation through adenoviral-mediated overexpression of OGT significantly suppresses ER stress and subsequent cell death in myocytes [69]. Similar results have been demonstrated in a rabbit model of renal I/R injury, where CHOP levels were suppressed upon administration of GlcN [70]. Although it is not yet clear how *O*-GlcNAc regulates ER stress, several mechanisms have been proposed. First, *O*-GlcNAcylation of eIF2α can inhibit phosphorylation of eIF2α, which is necessary for CHOP activation [71]. Second, OGT can suppress ER stress by stabilizing hypoxia-inducible factor 1α (HIF1α) [72]. Third, it has been proposed that *O*-GlcNAc plays a role in mitigating ER stress through formation of stress granules and processing bodies, which regulate mRNA translation and degradation, respectively [73]. While the functional contributions of CSD-induced ER stress and GSK-3β activation to CSD-induced AD pathogenesis, as well as the underlying mechanisms, remain to be elucidated, we tentatively suggest that induction of GSK‐3β activation or ER stress by CSD may not be the critical, sole effector of AD pathogenesis or a direct regulatory target of HBP/*O*-GlcNAc cycling under CSD conditions. As evidence for this, inhibition of neuroinflammation by minocycline did not affect either GSK‐3β activation or ER stress induced by CSD, despite the fact that it effectively rescued CSD-induced decreases in *O*-GlcNAc and AD pathogenesis as well as glial activation. Our study exclusively utilized male mice to minimize experimental variations caused by hormonal effects, aiming to achieve clearer results. However, this approach limits our understanding of potential sex differences in neurobiology and disease mechanisms, as well as the clinical applicability of our findings. Future studies should include validation using female mice to ensure the robustness and broader applicability of our research.

In the current study, pathogenic changes in the brain resulting from repeated CSD exposure are evident in our mice model, and the injury pattern shares some overlapping features with those of AD. The primary significance of our study lies in its pioneering proposal of a direct association between *O*-GlcNAc cycling in the brain during CSD and the functional and molecular neuronal changes induced by CSD. Future studies investigating the regulatory mechanisms governing HBP flux during sleep cycles, as well as the cyclic patterns of OGA and OGT expression, will be crucial in unraveling the association between sleep disorders and the development of AD.

### Electronic supplementary material

Below is the link to the electronic supplementary material.

Supplementary Material 1

Supplementary Material 2

## Data Availability

No datasets were generated or analysed during the current study.
