# Supplementary figures and images for: Chronic rapid eye movement sleep deprivation aggravates the pathogenesis of Alzheimer’s disease by decreasing brain O-GlcNAc cycling in mice
Source: J Neuroinflammation. 2024 Jul 23;21:180. doi: 10.1186/s12974-024-03179-4 (PMC11264383; doi:10.1186/s12974-024-03179-4)

Figure 3

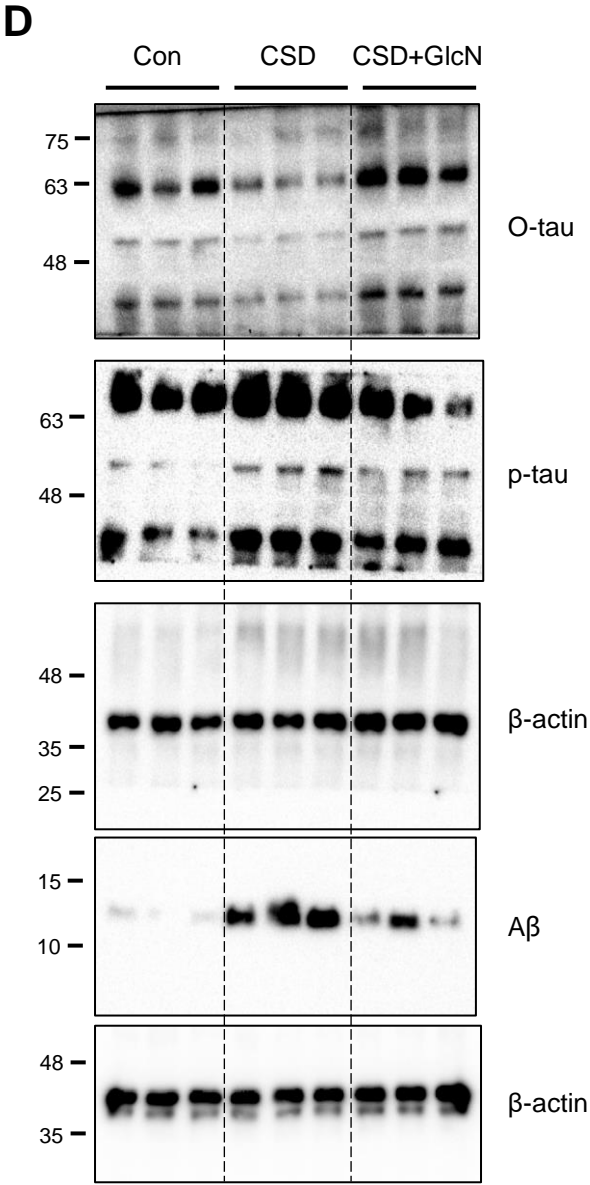

**Figure 4**

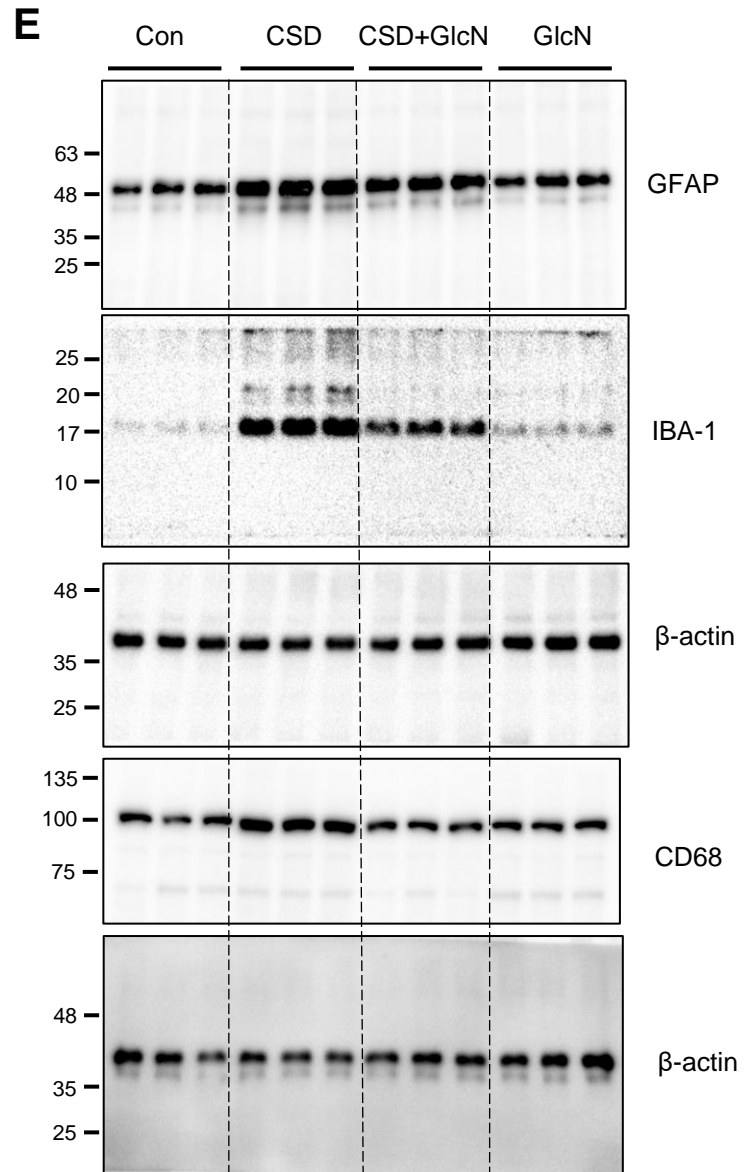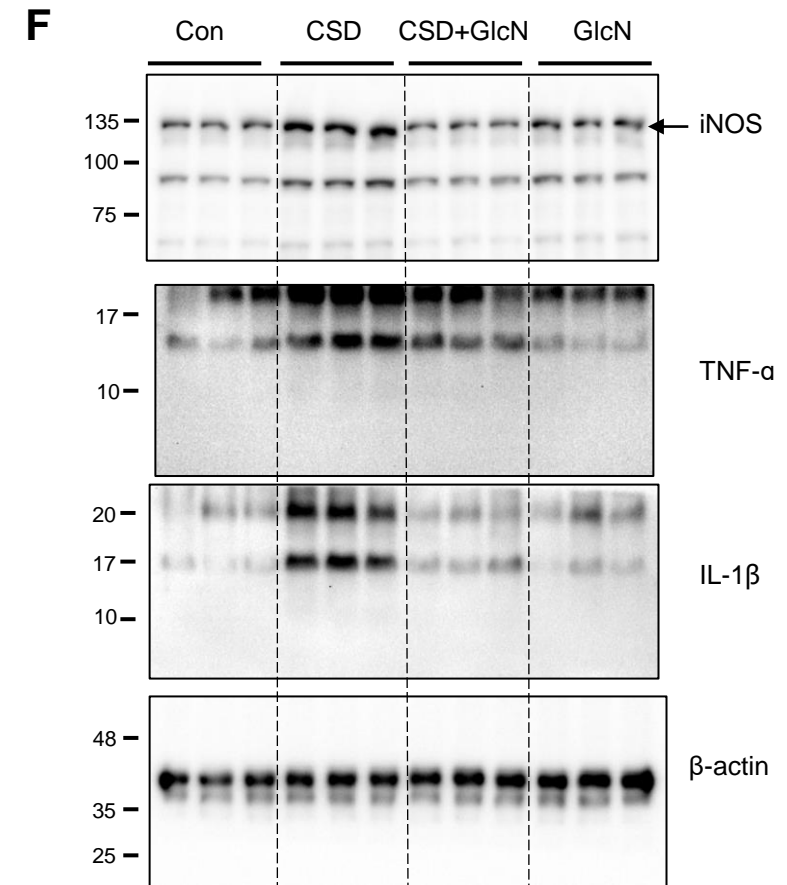

**A**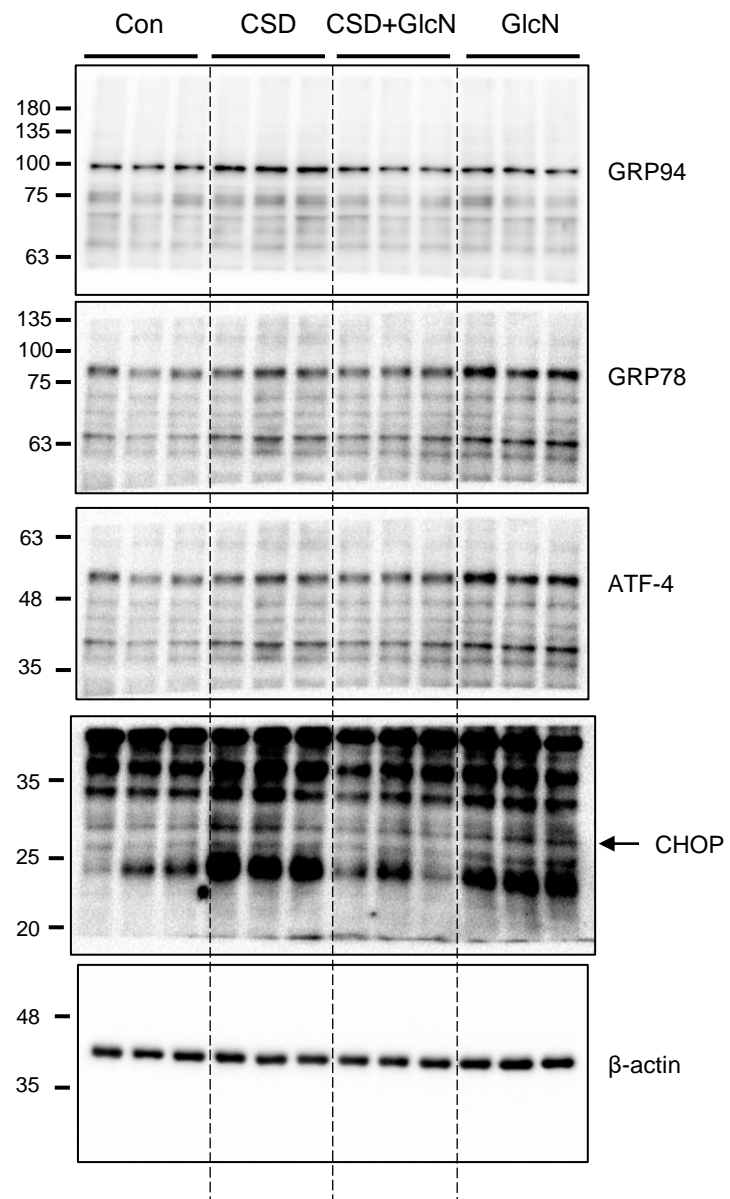**Figure 5****B**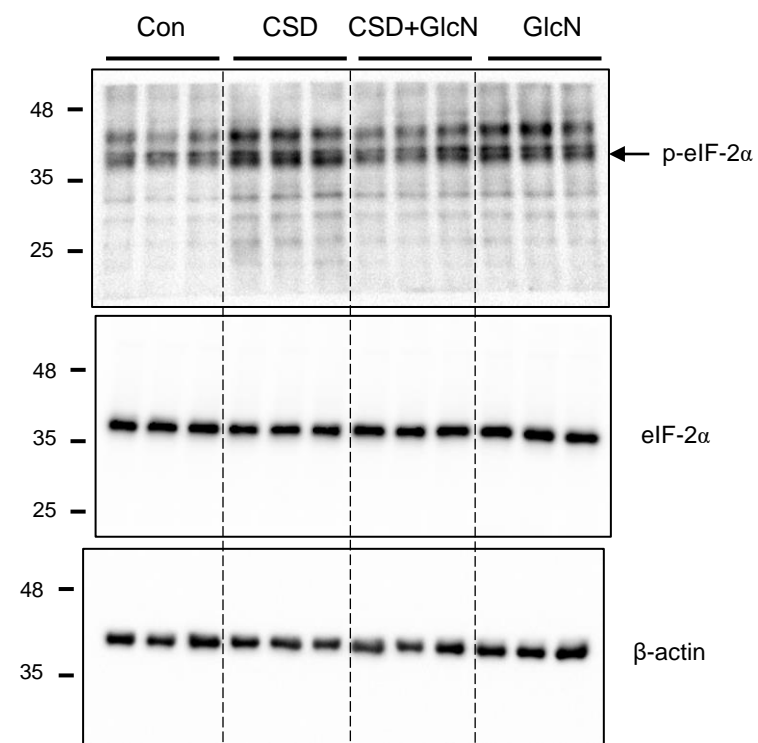

Figure 5

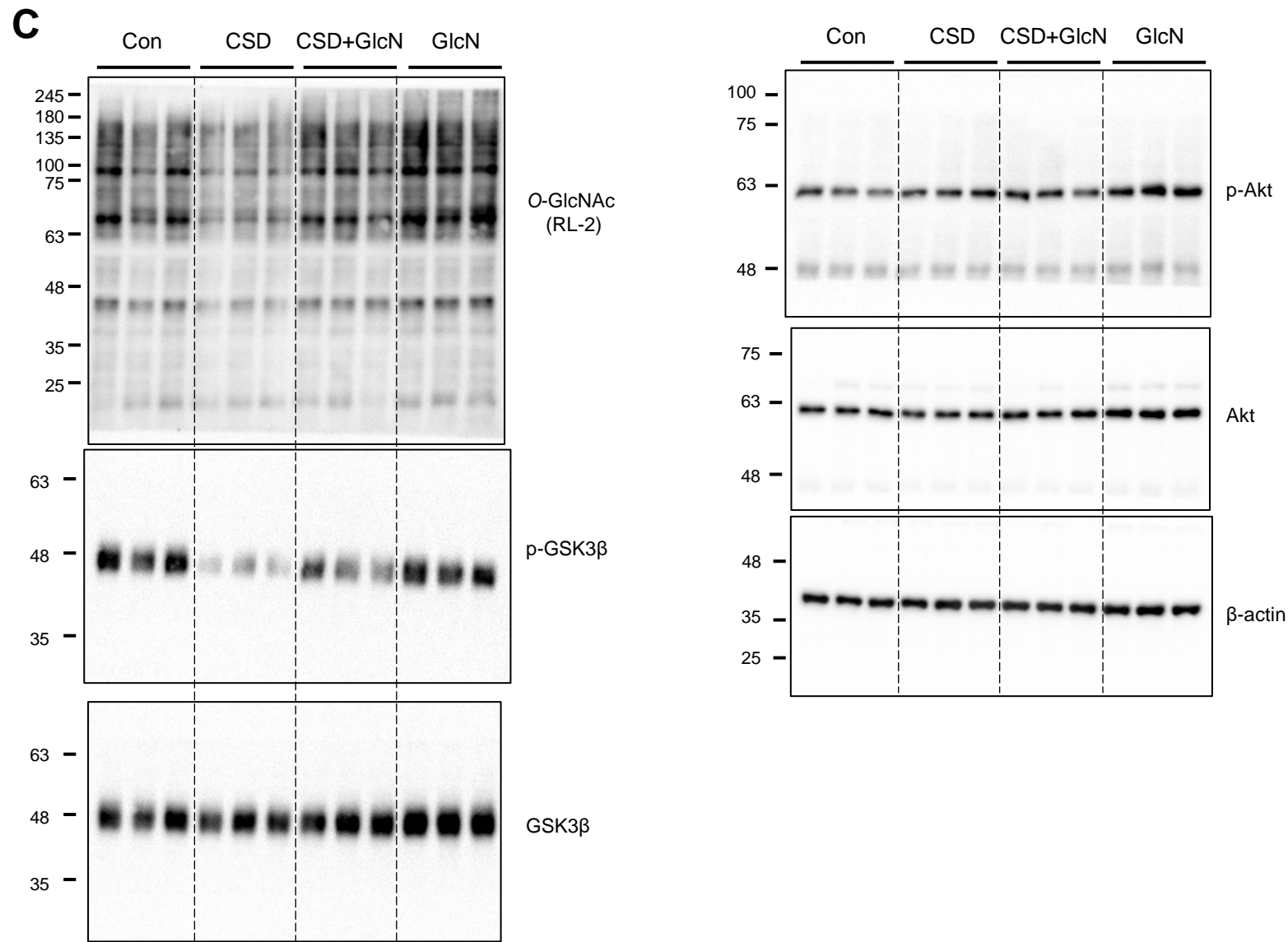

**Figure 5**

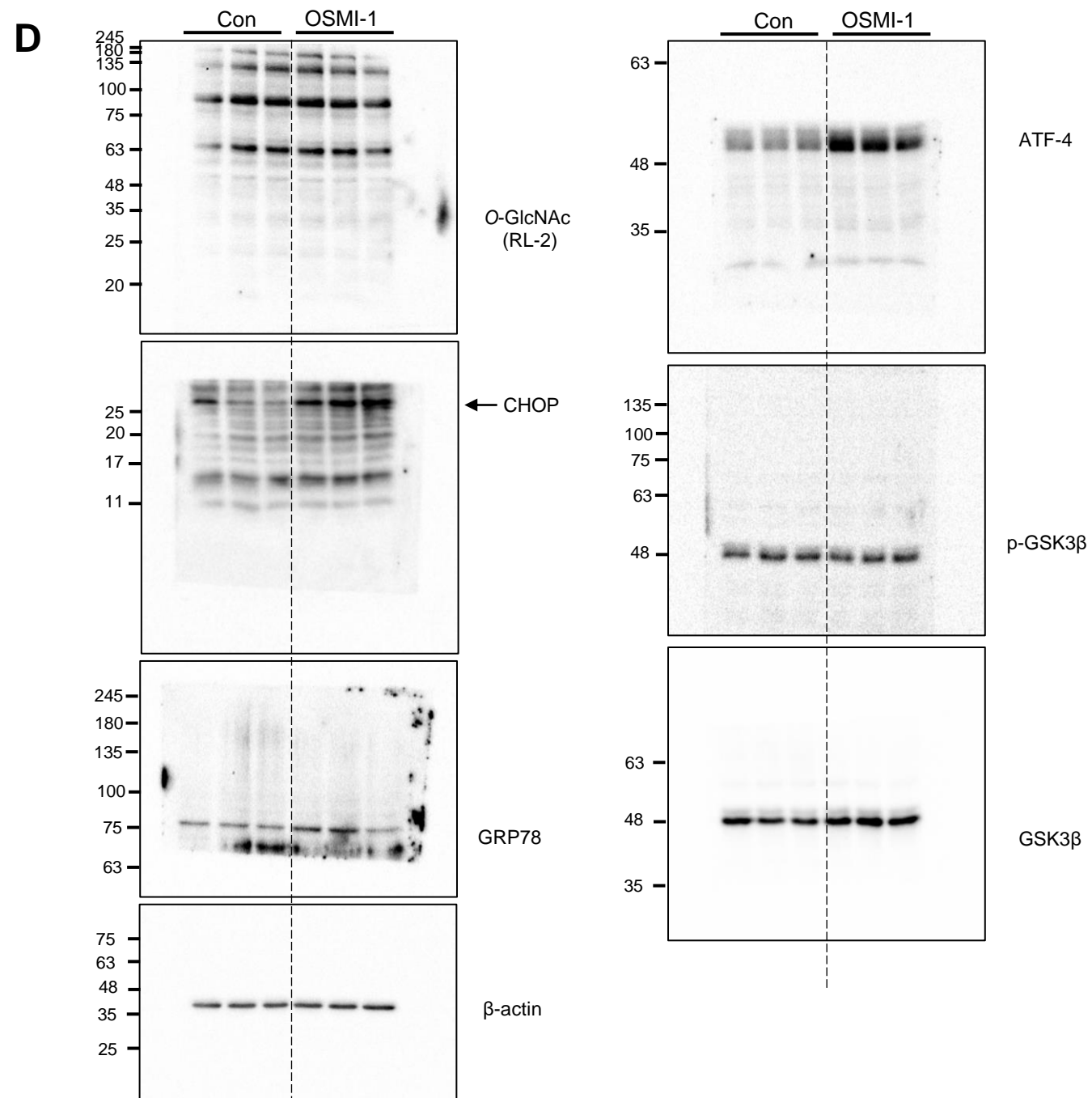

**Figure 7**

**A**

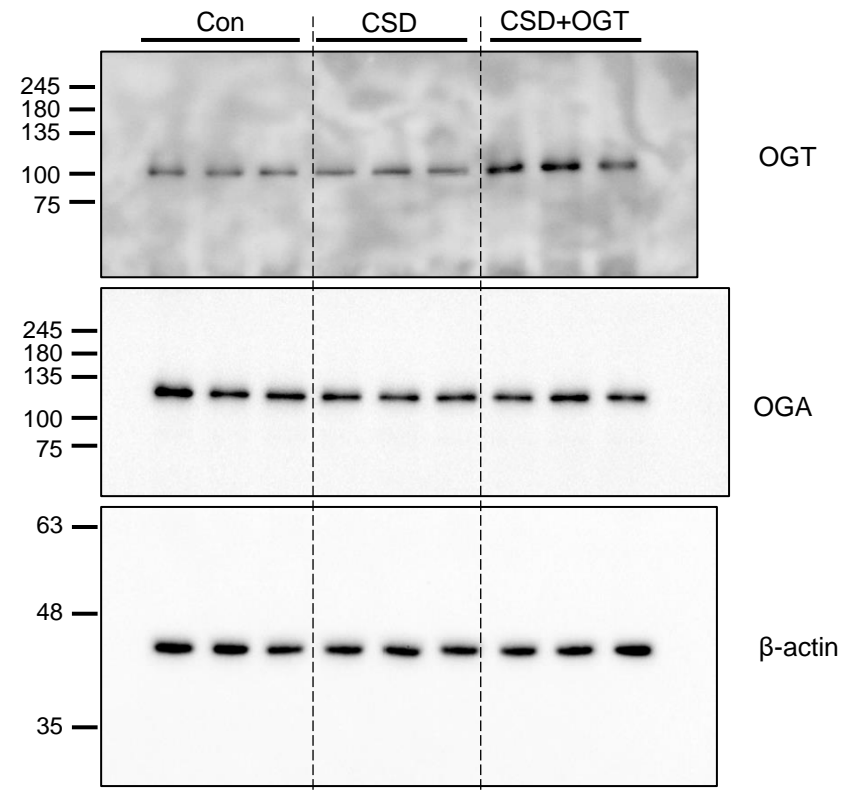

Figure 8

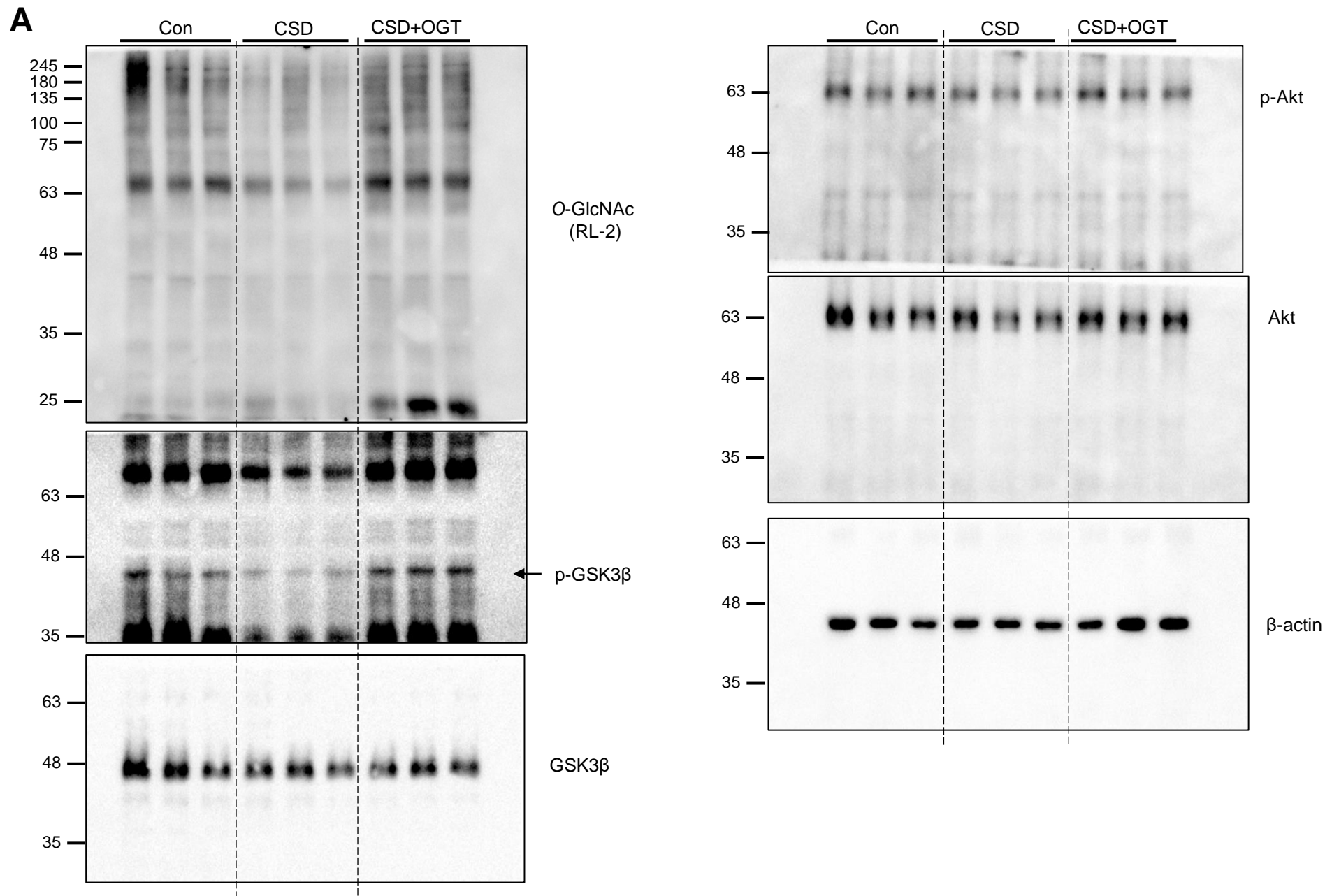

**B**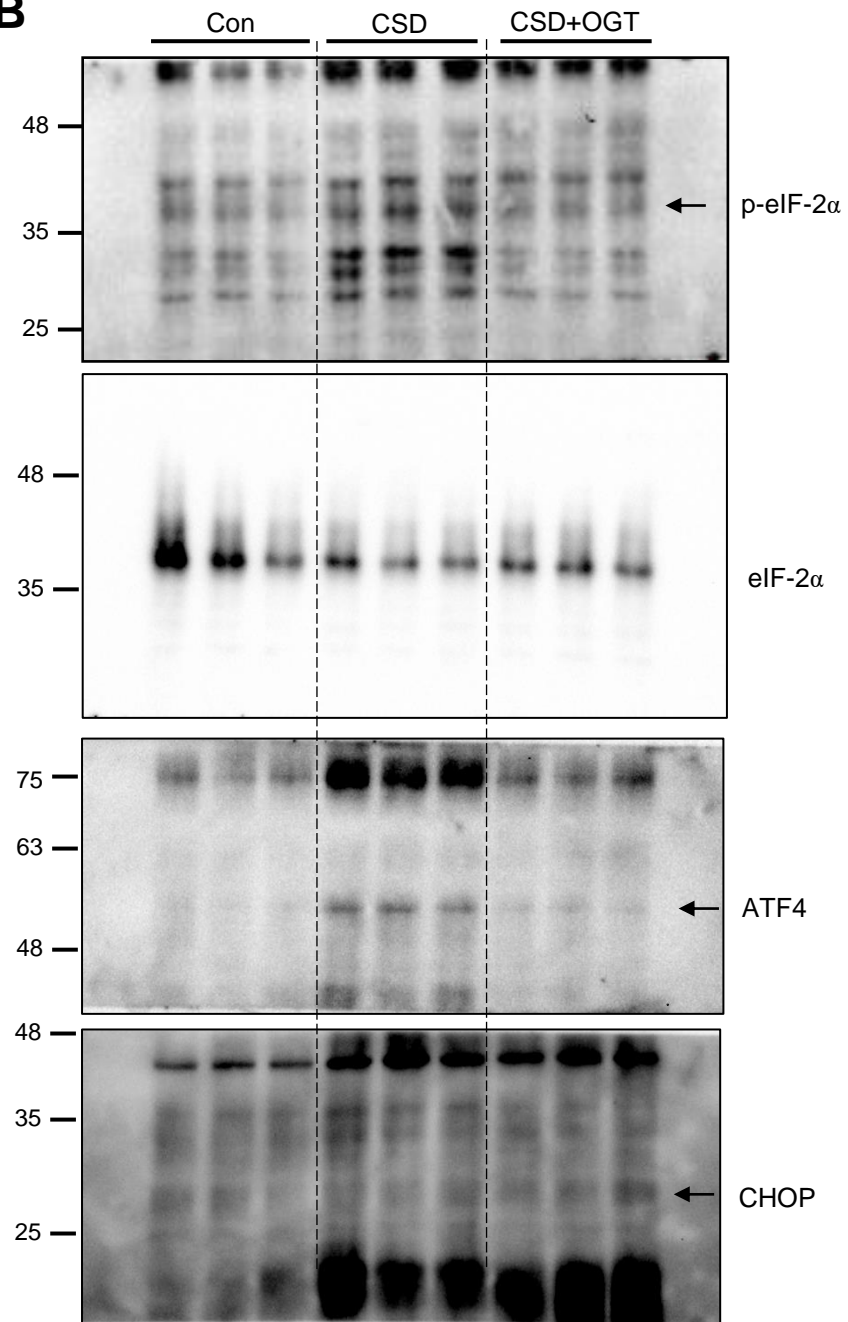**Figure 8**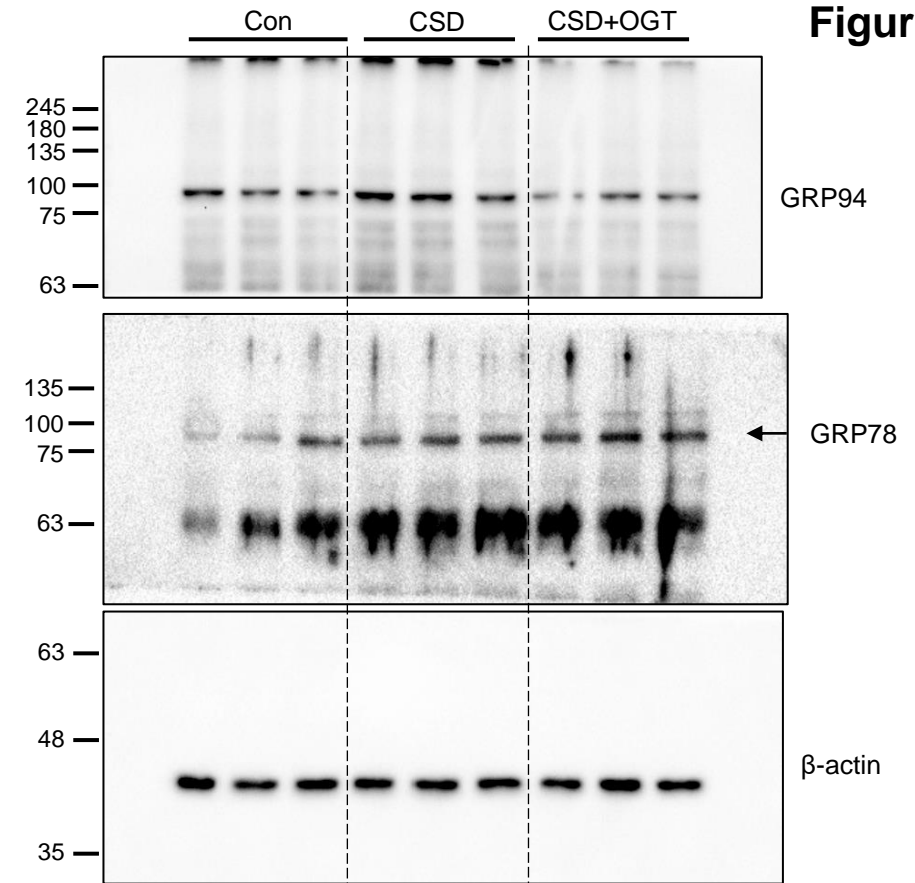

Supplement: Supplementary file 1 — Supplementary Material 1 [file 12974_2024_3179_MOESM1_ESM.pdf]
