# Supplementary material for: Chronic rapid eye movement sleep deprivation aggravates the pathogenesis of Alzheimer’s disease by decreasing brain O-GlcNAc cycling in mice
Source: J Neuroinflammation. 2024 Jul 23;21:180. doi: 10.1186/s12974-024-03179-4 (PMC11264383; doi:10.1186/s12974-024-03179-4)
Supplement: Supplementary file 2 — Supplementary Material 2 [file 12974_2024_3179_MOESM2_ESM.pdf]

Suppl. Figure 1

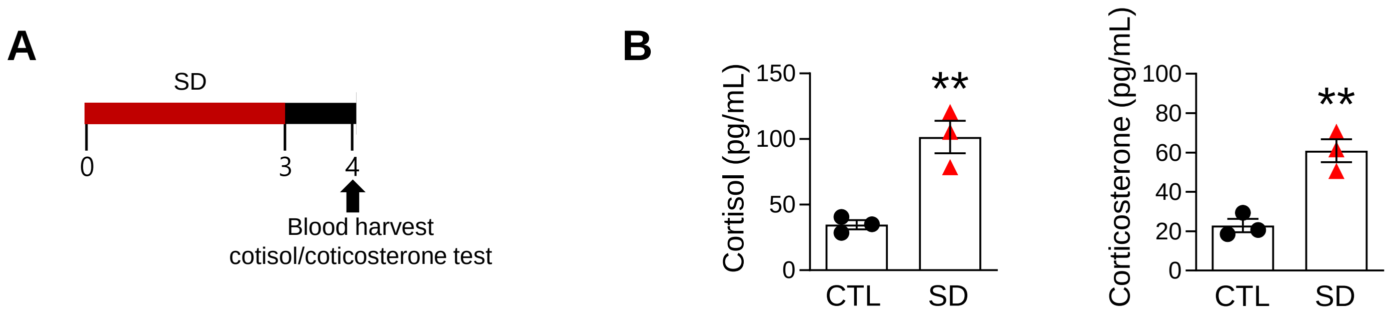

Suppl. Figure 2

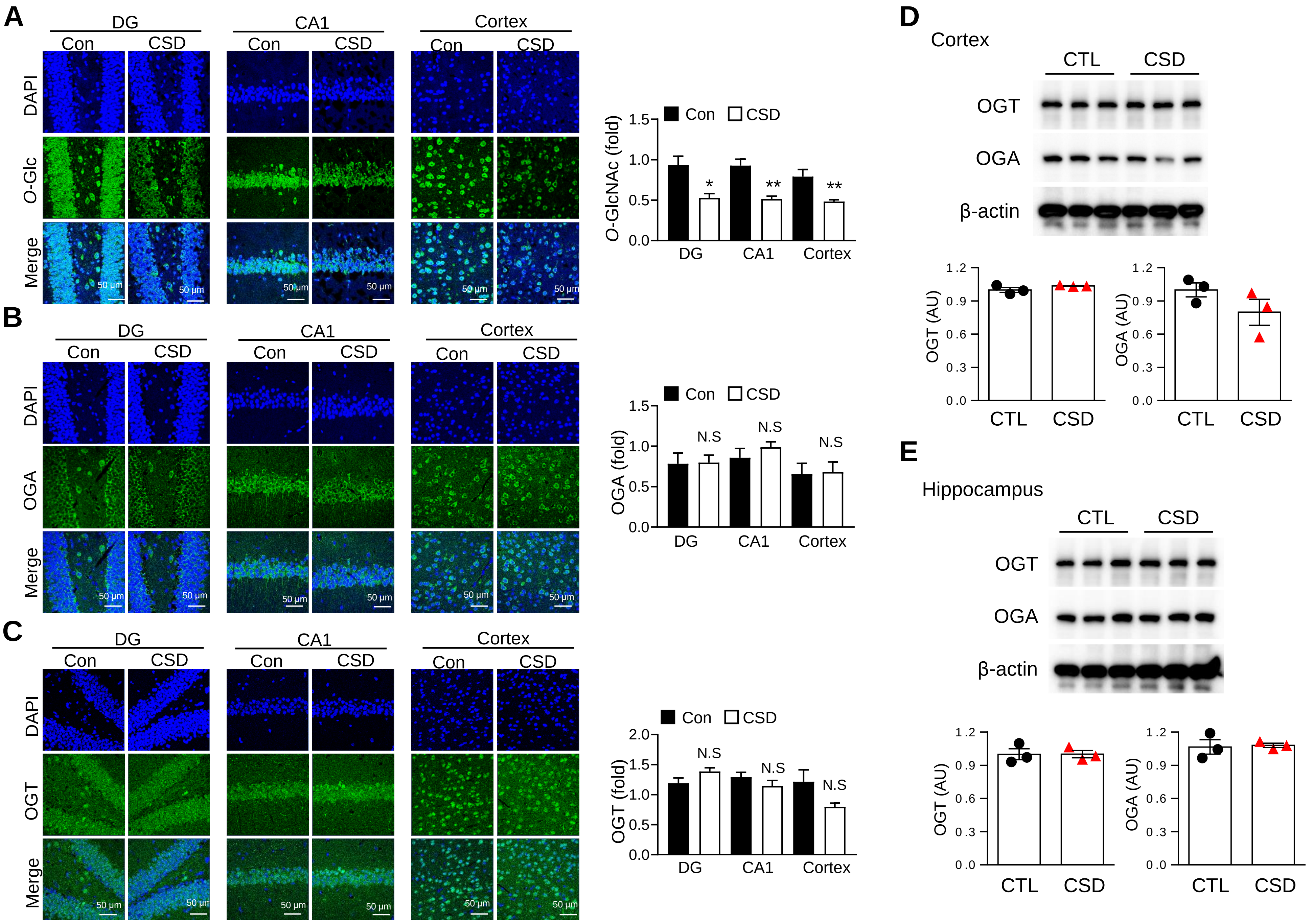

Suppl. Figure 3

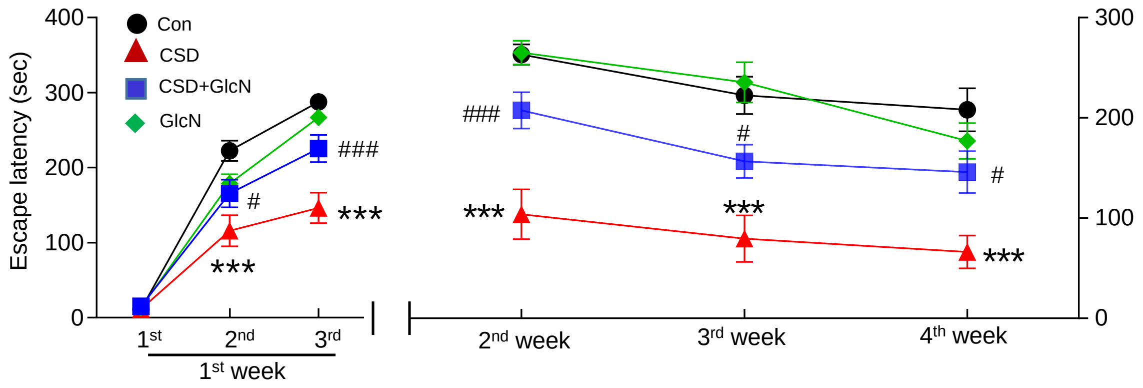

Suppl. Figure 4

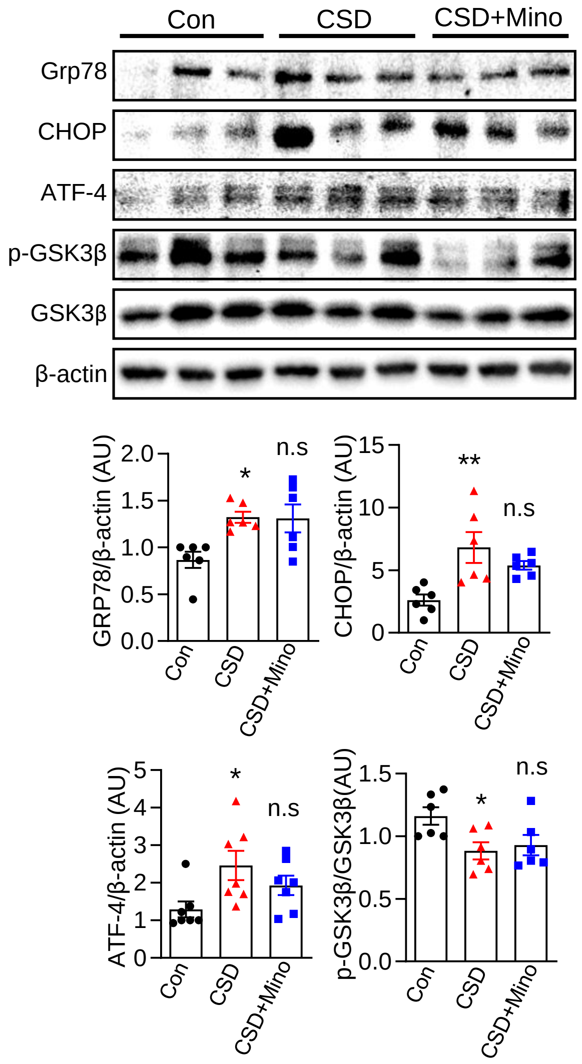

Supplementary Figure 1. Cortisol and corticosterone are increased by sleep deprivation. The multiple platform method, REMSD was induced in mice for 72 h. (A) Diagram of the experimental procedure for cortisol/corticosterone testing. (B) Graph showing plasma cortisol and corticosterone levels (n=3/group). Values are presented as mean ± SEM. Statistical analysis was conducted with Student's *t* test; $^{**}p < 0.05$ vs control group

Supplementary Figure 2. Effects of CSD on *O*-GlcNAc, OGA, and OGT levels in the mouse brain.

(A, B, C) Representative immunofluorescent staining (40X) of *O*-GlcNAc (A), OGA (B), OGT (C), and merged images in the brains of control and CSD mice is shown. DAPI staining was performed for nuclei visualization. The scale bar represents 50 μm. The graphs depict the quantification of *O*-GlcNAc, OGA, and OGT (n = 4/group), with values presented as the mean ± SEM. Statistical analysis was conducted using a Student's t-test; $^{*}p < 0.05$, $^{**}p<0.01$ vs control group, n.s: no significant. CSD, chronic sleep deprivation; Con, control; *O*-Glc, *O*-GlcNAc; OGA, *O*-GlcNAcase; OGT, *O*-GlcNAc transferase. (D, E) Representative western blot images of OGT, OGA and β-actin of hippocampus and cortex of mice brain. Graphs represent densitometric quantification of OGT, OGA, normalized by β-actin (n=3/group).

Supplementary Figure 3. The effect of GlcN on L/M dysfunction in CSD mice

The graph represents the escape latency during learning trials of the first (T1), second (T2), and third (T3) passive avoidance tests at one-hr intervals after the first cycle of SD. The memory test conducted after the second (M1), third (M2), and fourth (M3) cycle during CSD.

Supplementary Figure 4. Effects of minocycline on induction of ER molecules and GSK3β activation

Representative western blot images of GRP78, CHOP, ATF-4, p-GSK3β, GSK3βand β-actin of hippocampus of mice brain. Graphs represent densitometric quantification of GRP78, CHOP, ATF-4, normalized by β-actin and p-GSK3β, normalized by GSK3β (n = 6/group). The values are presented as mean ± SEM.;$^{*}p<0.05$, $^{**}p<0.01$, vs control group, n.s: no significant.
